# T cell metabolic reprogramming in acute kidney injury and protection by glutamine blockade

**DOI:** 10.1172/jci.insight.160345

**Published:** 2023-06-22

**Authors:** Kyungho Lee, Elizabeth A. Thompson, Sepideh Gharaie, Chirag H. Patel, Johanna T. Kurzhagen, Phillip M. Pierorazio, Lois J. Arend, Ajit G. Thomas, Sanjeev Noel, Barbara S. Slusher, Hamid Rabb

**Affiliations:** 1 Department of Medicine, Johns Hopkins University School of Medicine, Baltimore, Maryland, USA.; 2 Nephrology Division, Department of Medicine, Samsung Medical Center, Cell and Gene Therapy Institute, Sungkyunkwan University School of Medicine, Seoul, South Korea.; 3 Department of Oncology,; 4 Bloomberg~Kimmel Institute for Cancer Immunotherapy,; 5 Department of Urology,; 6 Department of Pathology, and; 7 Department of Neurology and Drug Discovery Program, Johns Hopkins University School of Medicine, Baltimore, Maryland, USA.

**Keywords:** Immunology, Nephrology, T cells

## Abstract

T cells play an important role in acute kidney injury (AKI). Metabolic programming of T cells regulates their function, is a rapidly emerging field, and is unknown in AKI. We induced ischemic AKI in C57BL/6J mice and collected kidneys and spleens at multiple time points. T cells were isolated and analyzed by an immune-metabolic assay. Unbiased machine learning analyses identified a distinct T cell subset with reduced voltage-dependent anion channel 1 and mTOR expression in post-AKI kidneys. Ischemic kidneys showed higher expression of trimethylation of histone H3 lysine 27 and glutaminase. Splenic T cells from post-AKI mice had higher expression of glucose transporter 1, hexokinase II, and carnitine palmitoyltransferase 1a. Human nonischemic and ischemic kidney tissue displayed similar findings to mouse kidneys. Given a convergent role for glutamine in T cell metabolic pathways and the availability of a relatively safe glutamine antagonist, JHU083, effects on AKI were evaluated. JHU083 attenuated renal injury and reduced T cell activation and proliferation in ischemic and nephrotoxic AKI, whereas T cell–deficient mice were not protected by glutamine blockade. In vitro hypoxia demonstrated upregulation of glycolysis-related enzymes. T cells undergo metabolic reprogramming during AKI, and reconstitution of metabolism by targeting T cell glutamine pathway could be a promising novel therapeutic approach.

## Graphical abstract

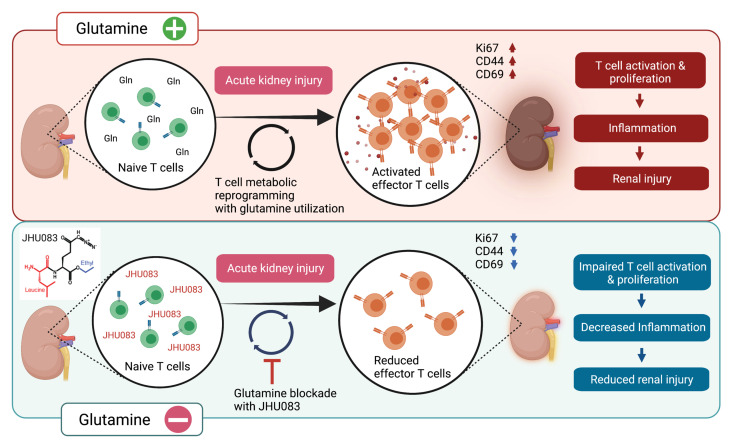

## Introduction

Acute kidney injury (AKI) is an important clinical problem affecting both native kidneys and renal allografts. There is no specific treatment for AKI except for supportive measures including fluid therapy or dialysis (1). There are many cellular and molecular mechanisms involved in AKI pathogenesis, including inflammation, epigenetics, cell death pathways, epithelial cell metabolic abnormalities, and others (2–7). T cells are established to play a modulatory role in AKI and repair (8, 9).

Metabolic reprogramming has emerged as a central mechanism of T cell activation and differentiation (10). Metabolic pathways such as glutaminolysis, glycolysis, fatty acid oxidation, and oxidative phosphorylation (OXPHOS) and their metabolites were traditionally considered downstream consequences of cellular function (11). However, there is an increasing recognition that these metabolic pathways play important and coordinated roles as regulators promoting differentiation and activation of T cells (11–13). During the past decade, there have been substantial advances in understanding T cell metabolic programming through various disease models (14–17), but this has not been studied in the context of AKI. T cells are predicted to undergo metabolic reprogramming during AKI since it is known that their numbers increase and are activated early during AKI (18, 19). Furthermore, considering that ischemic AKI results from exposure to hypoxia followed by reoxygenation, kidney T cells may use alternative energy sources during the ischemia and after reperfusion to maintain their effector function.

Reconstitution of metabolic pathways using specific inhibitory molecules can have immunomodulatory effects (14, 20, 21). Given the high metabolic demands of effector T cells, blocking metabolic pathways can affect T cells selectively, not altering many normal cellular homeostatic functions, which have more metabolically flexible mechanisms (14, 22). Among metabolic pathways, the glutaminolysis pathway is among the important metabolic checkpoints in T cells, since activated T cells utilize aerobic glutamine metabolism to fuel high proliferative rates (23). Given the important role of T cells as an early responder in AKI (9), modulation of the glutamine pathway using a specific inhibitory agent could modify T cell function and influence renal outcomes after AKI.

We hypothesized that T cells undergo metabolic reprogramming during experimental AKI. To study the metabolic landscape of T cells, we used spectral flow cytometry–based immune-metabolic assay along with unsupervised computational analyses to identify metabolically dysregulated T cell subsets in postischemic mouse kidneys (24). We then studied human samples to determine clinical relevance. After finding pronounced and broad changes in T cell metabolism during AKI, we chose a select metabolic pathway, glutamine utilization, where many of the abnormalities converged and an interventional agent was available with efficacy and safety in cancer models. Glutamine blockade was performed with the glutamine antagonist JHU083 in both ischemic and nephrotoxic AKI models in mice. We found that the JHU083 treatment changed kidney T cells to naive phenotype and improved functional and structural renal injury. To further identify mechanisms of JHU083 action, we studied the effects of JHU083 on in vitro hypoxia followed by reoxygenation of T cells. Our findings demonstrate key T cell metabolic changes in murine and human AKI and that reconstitution of T cell metabolism in AKI could be a novel therapeutic strategy for AKI.

## Results

### Immune-metabolic assay and metabolic signature of activated T cells.

**Figure 1:**
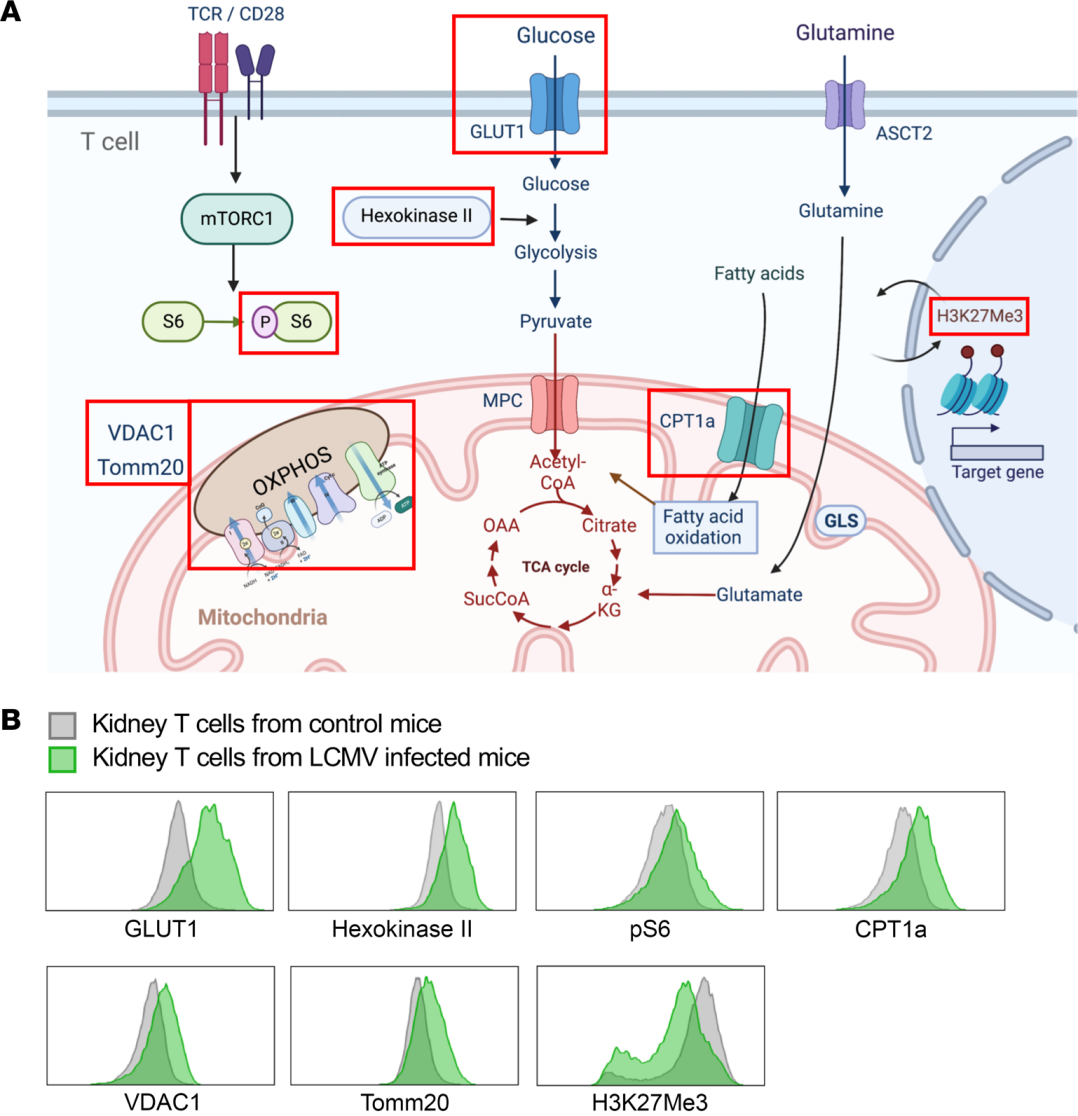
Metabolic pathways studied and metabolic signature of in vivo–activated kidney T cells. (**A**) We evaluated key enzymes for glycolysis, fatty acid oxidation, and mitochondrial oxidative phosphorylation, as well as a histone methylation marker. Glucose transporter 1 (GLUT1) and HKII (hexokinase II) were used to evaluate glycolysis machinery. CPT1a expression was measured for fatty acid oxidation. mTOR signaling activity was measured with S6 ribosomal protein phosphorylation. Mitochondrial oxidative phosphorylation was measured with VDAC1 and Tomm20. H3K27me3 was measured as a readout for histone methylation. (**B**) Histograms comparing kidney T cells from control mice (gray) and LCMV-infected mice (green) as a positive control (on day 7 after LCMV inoculation). ASCT2, alanine-serine-cysteine transporter 2; CPT1a, carnitine palmitoyltransferase 1a; GLS, glutaminase; H3K27me3, trimethylation of histone H3 lysine 27; LCMV, lymphocytic choriomeningitis virus; MPC, mitochondrial pyruvate carrier; pS6, phosphorylated ribosomal protein S6; VDAC1, voltage-dependent anion channel 1.

A spectral flow cytometry–based immune-metabolic assay was used to study T cell metabolic programs (Figure 1A). To assess the glycolytic machinery, we evaluated GLUT1 and the rate-limiting enzyme of glycolysis, HKII. Fatty acid oxidation was measured by expression of a rate-limiting enzyme, CPT1a. mTOR signaling activity was measured by p-S6. Mitochondrial OXPHOS was assessed using mitochondrial membrane proteins, VDAC1 and Tomm20. Since changes in cellular metabolism lead to epigenetic modulation of T cells through metabolites, we measured trimethylation of histone H3 lysine 27 (H3K27Me3), which is controlled by metabolites of the TCA cycle, as a readout for histone methylation (25). The gating strategies for kidney T cells are provided in Supplemental Figure 1; supplemental material available online with this article; https://doi.org/10.1172/jci.insight.160345DS1

To establish a system to measure the metabolic signature of activated T cells with a positive disease control, T cells from lymphocytic choriomeningitis virus–infected (LCMV-infected) mouse kidneys were analyzed on day 7 after virus inoculation and compared with those from normal control mice. LCMV is known to directly infect kidneys and activate T cells in a noncytopathic manner (26). Enzymes involved in glycolysis, OXPHOS, fatty acid oxidation, and mTOR activity were globally upregulated, whereas the repressive histone methylation marker H3K27me3 was downregulated in kidney T cells from LCMV-infected mice (Figure 1B).

### Postischemic kidneys reveal a distinct T cell subset with impaired mTOR and OXPHOS activity.

**Figure 2:**
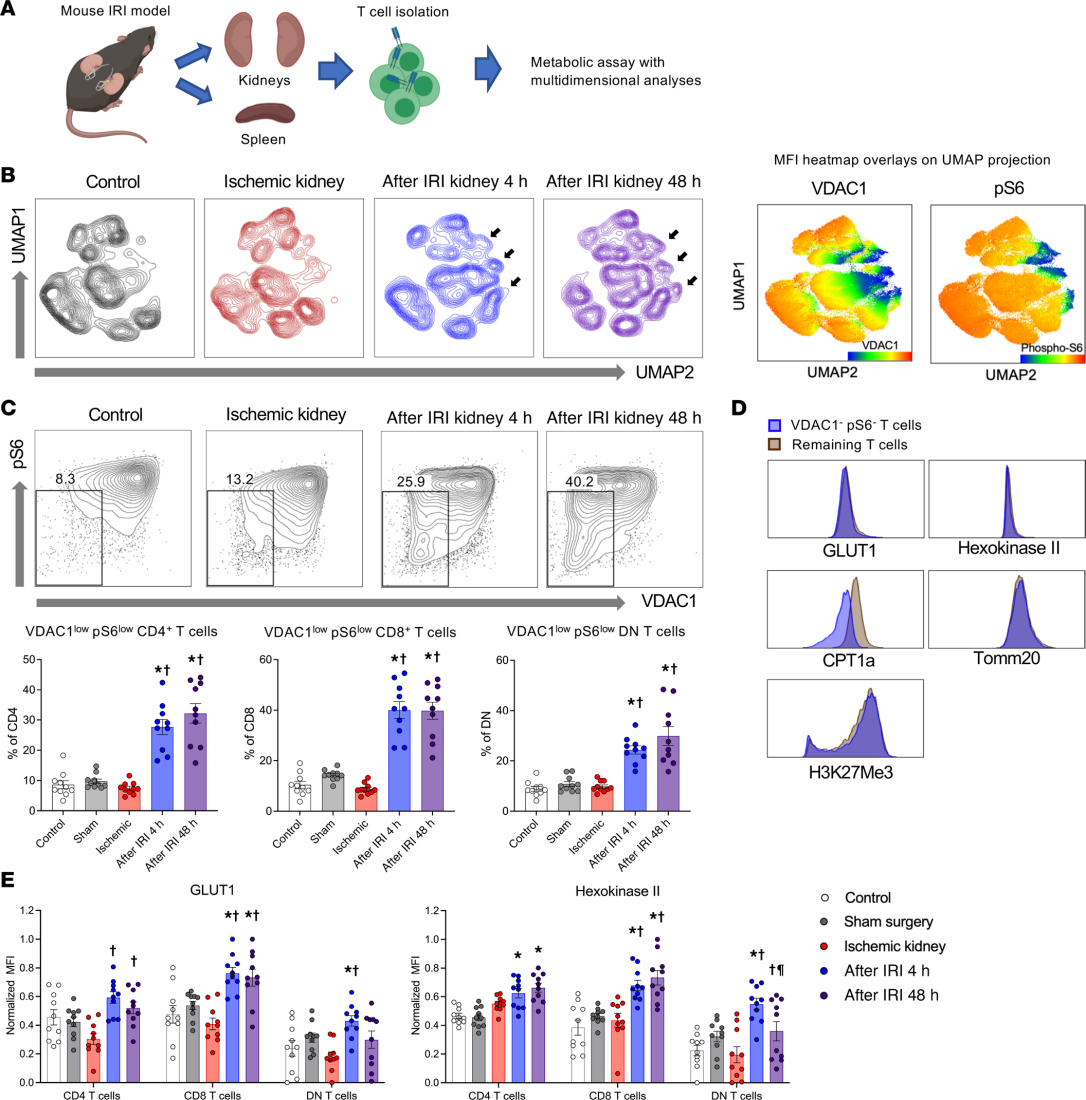
A metabolically distinct subset of T cells with low VDAC1 and low p-S6 expression increases in kidneys following ischemic AKI. (**A**) Schematic of the experimental design. (**B**) Concatenated flow cytometry data depicted as UMAP of T cells in control kidneys, ischemic kidneys, and post-IRI kidneys. Post-IRI kidneys showed distinct segregated populations having low VDAC1 and p-S6 expressions compared with the control kidneys and ischemic kidneys. (**C**) Representative flow plots showing VDAC1^lo^p-S6^lo^ T cells. Frequencies of VDAC1^lo^p-S6^lo^ subsets among CD4^+^, CD8^+^, and DN T cells in mouse kidneys. They were significantly increased following reperfusion. Statistical analyses were performed using 1-way ANOVA followed by Tukey’s post hoc analysis (*n* = 10 mice in each group). Data are from 2 independent experiments. (**D**) Histograms comparing VDAC1^lo^p-S6^lo^ T cells (blue) and remaining T cells (brown) from concatenated post-IRI 48 hours data. (**E**) Changes in glycolysis enzymes on VDAC1^lo^p-S6^lo^ T cells according to different time points. GLUT1 and HKII on those cells were increased significantly following IRI. Statistical analyses were performed using 2-way ANOVA followed by Tukey’s post hoc analysis (*n* = 10 mice in each group). Data are from 2 independent experiments. *P < 0.05, compared with the control group; †*P* < 0.05, compared with the ischemia group; ^¶^*P* < 0.05, compared with the post-IRI 4 hours group. CPT1a, carnitine palmitoyltransferase 1a; DN, double-negative; IRI, ischemia/reperfusion injury; MFI, mean fluorescence intensity; pS6, phosphorylated ribosomal protein S6; UMAP, uniform manifold approximation and projection; VDAC1, voltage-dependent anion channel 1.

To elucidate T cell metabolic reprogramming in ischemic AKI, we induced bilateral ischemia/reperfusion injury (IRI) for 30 minutes and procured kidneys at multiple early postreperfusion time points, including during ischemia, post-IRI 4 hours, and post-IRI 48 hours. Kidney T cells were isolated with an established technique (18) and analyzed with high-dimensional unbiased analyses using uniform manifold approximation and projection (UMAP) algorithm (Figure 2A).

Unsupervised multidimensional analyses revealed a distinct T cell population in postischemic kidneys, with low expression of mTOR activity, assessed by p-S6, and VDAC1 (Figure 2B). Based on this, we subsequently gated the VDAC1^lo^ and p-S6^lo^ population, and the percentages of this population were significantly increased in post-IRI kidneys compared with those from control kidneys and sham surgery kidneys (Figure 2C). The VDAC1^lo^p-S6^lo^ T cells were not limited to specific immunophenotypic populations, involving both effector memory and naive subsets of CD4^+^ and CD8^+^ T cells as well as DN T cells. These distinct T cell subsets expressed a lower level of CPT1a compared with the remaining T cells, but they maintained comparable levels of glycolytic enzyme expression (Figure 2D). This finding may indicate that T cells reduce OXPHOS and fatty oxidation under ischemic AKI and utilize glycolysis selectively. Within VDAC1^lo^p-S6^lo^ T cell subsets, GLUT1 and HKII had upregulated expression after reperfusion compared with in control kidneys (Figure 2E).

### Glutaminase activity was upregulated during ischemia.

**Figure 3:**
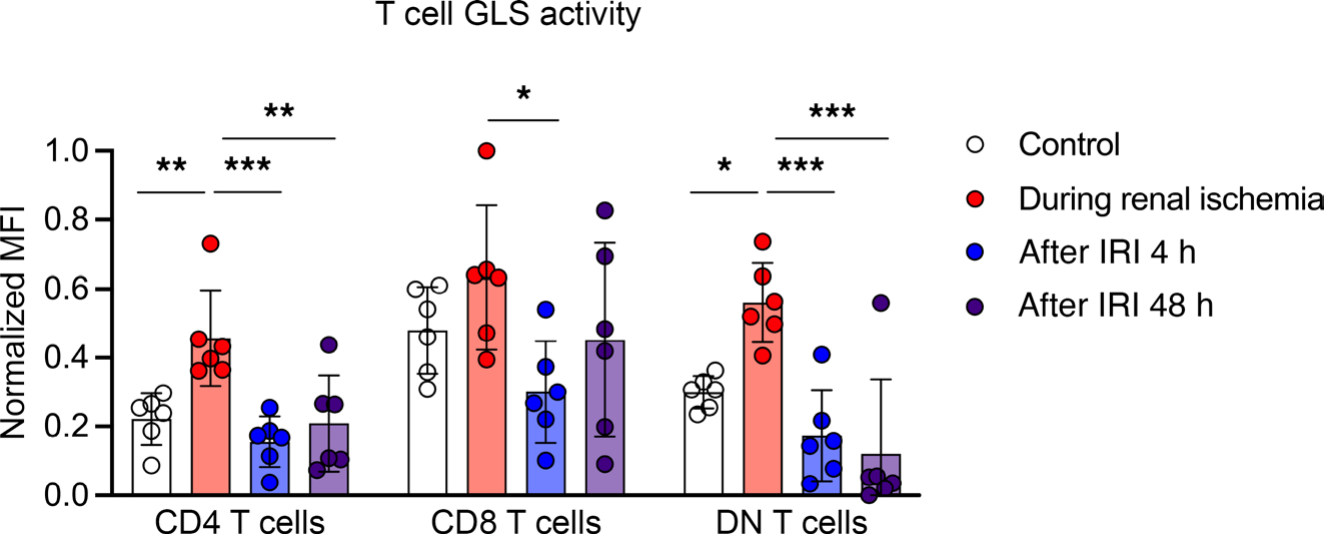
Kidney T cell glutaminase activity in ischemia AKI. Glutaminase activity significantly increased in T cells during ischemia. Statistical analyses were performed using 2-way ANOVA followed by Tukey’s post hoc analysis (*n* = 6 mice in each group). *P < 0.05; **P < 0.01; ***P < 0.001. DN, double-negative; GLS, glutaminase; IRI, ischemia/reperfusion injury; MFI, mean fluorescence intensity.

T cells during ischemia showed upregulated glutaminase activities compared with the control kidneys, and they decreased after reperfusion (Figure 3).

### Metabolic reprogramming of splenic T cells in ischemic AKI.

**Figure 4:**
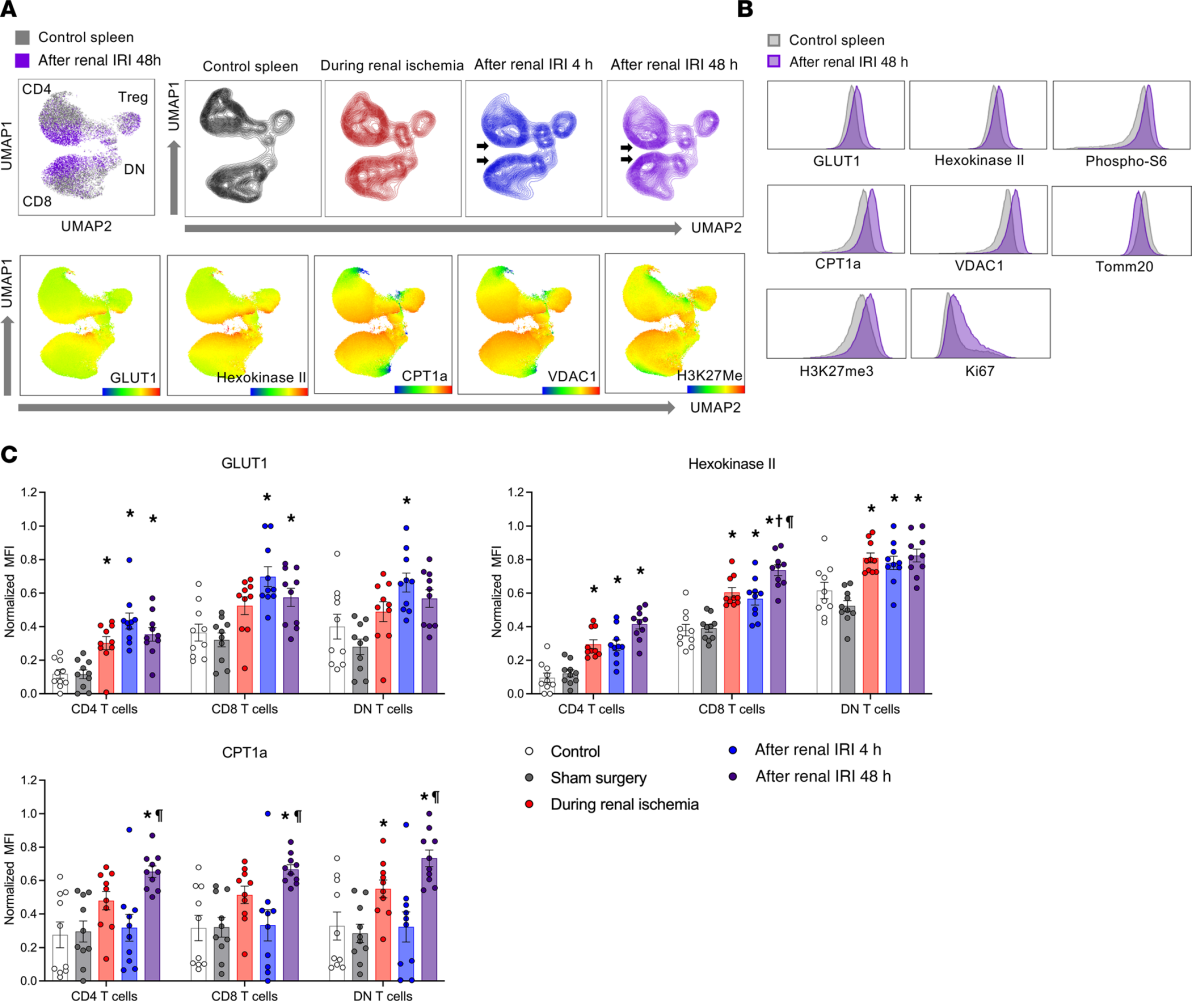
Splenic T cells have higher metabolic activity following ischemic AKI. (**A**) Unbiased UMAP analysis of concatenated flow cytometry data of splenic T cells from control mice (gray and black), mice during renal ischemia (red), and post-renal IRI 4 hours (blue) and 48 hours (purple) mice. Multiple enzymes associated with glycolysis, fatty acid oxidation, and oxidative phosphorylation drove the separation of splenic T cells, showing that metabolically activated T cell subsets were increased in the spleens following renal IRI (arrows). (**B**) Histograms comparing splenic T cells from control mice (gray) and post-IRI mice (48 hours) (purple). (**C**) Normalized MFI of significantly changed metabolic markers on splenic CD4^+^, CD8^+^, and DN T cells. Statistical analyses were performed using 2-way ANOVA followed by Tukey’s post hoc analysis (*n* = 10 mice in each group). Data are from 2 independent experiments. *P < 0.05, compared with the control group; †*P* < 0.05, compared with the renal ischemia group; ^¶^*P* < 0.05, compared with the post-renal IRI 4 hours group. AKI, acute kidney injury; CPT1a, carnitine palmitoyltransferase 1a; DN, double-negative; IRI, ischemia/reperfusion injury; UMAP, uniform manifold approximation and projection.

Given that AKI has a systemic immunologic effect (27), splenic T cells isolated from mice that underwent renal IRI were also studied at multiple time points. It is also easier to isolate and study the large number of T cells in spleens than kidneys. Unbiased multidimensional analyses showed higher expression of GLUT1, HKII, CPT1a, VDAC1, and H3K27Me3 in post-IRI mice compared with the control mice (Figure 4A). Splenic T cells from post-IRI mice exhibited phenotypes indicative of higher metabolic activity compared with those from the control and sham surgery mice with higher expression of GLUT1, HKII, and CPT1a, indicating an upregulation of glycolysis and fatty acid oxidation machinery (Figure 4, B and C). Since T cells in spleens are not directly exposed to metabolic stress from ischemia, unlike kidney T cells, these findings demonstrate the remote immunologic effect of T cell metabolism during AKI.

### In vitro hypoxia leads to upregulation of glycolysis in kidney T cells.

**Figure 5:**
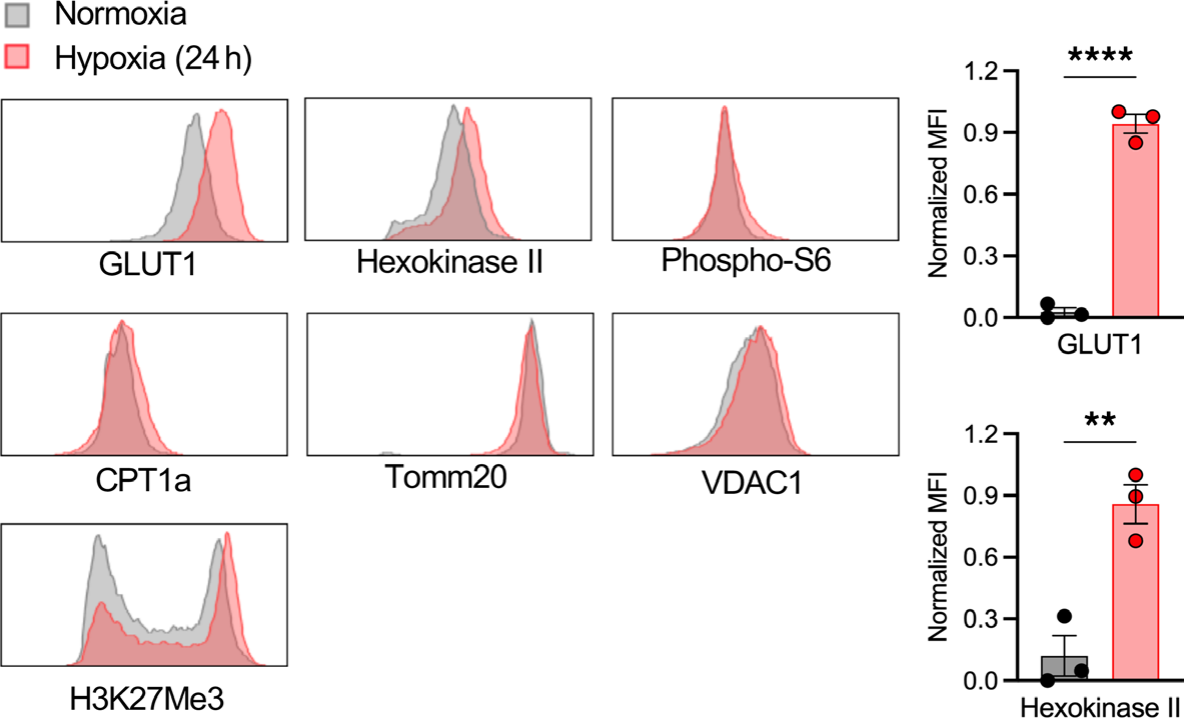
Effect of in vitro hypoxia on kidney T cell metabolism. FACS-sorted kidney T cells were cultured under CD3/CD28 stimulation and exposed to hypoxia for 24 hours followed by reoxygenation. T cells exposed to hypoxia showed higher levels of HKII and GLUT1 expression. Statistical analyses were performed using 2-tailed *t* test. *P < 0.05; **P < 0.01.

To test the effect of in vitro hypoxia followed by reoxygenation on activated T cell metabolism, FACS-sorted kidney T cells (CD45^+^, TCRαβ^+^) were cultured with CD3/CD28 stimulation and incubated in a hypoxic chamber for 24 hours followed by reoxygenation under normoxia for 24 hours. T cells exposed to hypoxia showed upregulation of enzymes involved in glycolysis machinery, including GLUT1 and HKII, compared with T cells under normoxic condition (Figure 5). The in vitro anaerobic environment appeared to induce enhanced glycolysis in activated kidney T cells. However, unlike in vivo data, the other enzymes were not downregulated following hypoxia exposure.

### H3K27me3 expression distinguishes T cells in nonischemic and ischemic human kidneys.

**Figure 6:**
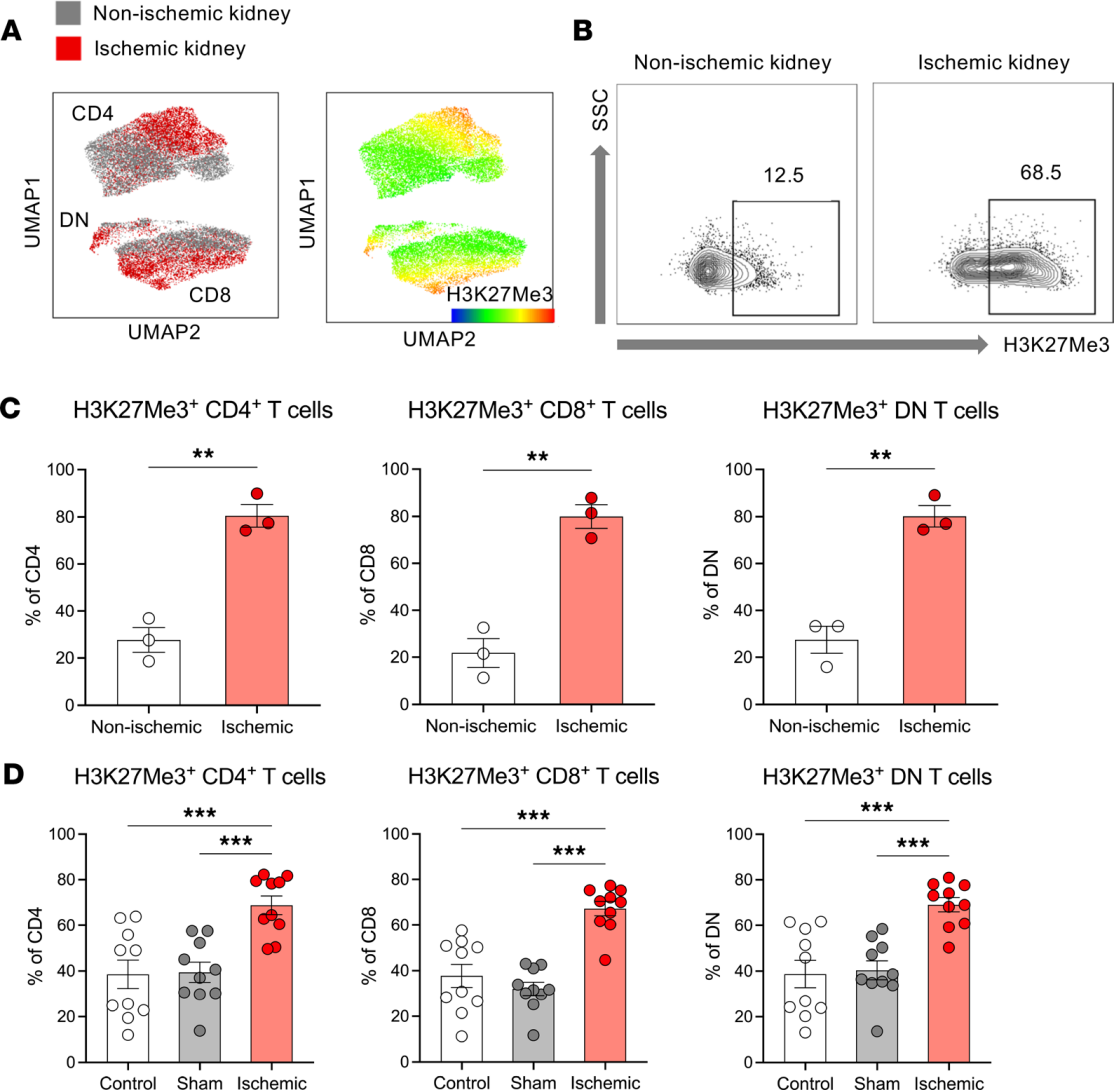
H3K27Me3^+^ T cells increase in human kidneys during ischemia. (**A**) Concatenated flow cytometry data depicted as UMAP of T cells from human nonischemic kidneys (gray) and ischemic kidneys (red). H3K27Me3 expression drove separation of the nonischemic and ischemic cluster. (**B**) Representative flow plots of human nonischemic kidney and ischemic kidneys depicting expression of H3K27Me3^+^. (**C**) Frequencies of H3K27Me3^+^ subsets among CD4^+^, CD8^+^, and DN T cells in human kidneys. T cells in ischemic kidneys showed significantly higher frequencies of H3K27Me3^+^ cells compared with those of nonischemic kidneys. Statistical analyses were performed using 2-tailed *t* test (*n* = 3 in each group for humans). (**D**) Frequencies of H3K27Me3^+^ subsets among CD4^+^, CD8^+^, and DN T cells in mouse kidneys, showing consistent findings with human data. Statistical analyses were performed using 1-way ANOVA followed by Tukey’s post hoc analysis (*n* = 10 in each group for mice). Mouse data are from 2 independent experiments. *P < 0.05; **P < 0.01; ***P < 0.001. DN, double-negative; SSC, side scatter; UMAP, uniform manifold approximation and projection.

To evaluate human kidney T cell metabolic reprogramming in ischemic AKI, kidney tissues were obtained from patients who underwent nephrectomy for localized renal cell carcinoma. Nonischemic (before clamping of renal artery) and ischemic kidney tissues (after clamping of renal artery) were procured separately from grossly “normal” portions of kidneys. In unbiased multidimensional analyses, upregulated H3K27Me3 expression drove the separation of ischemic kidney T cells from nonischemic kidneys (Figure 6A). The proportion of H3K27Me3^+^ T cell subsets was higher in ischemic kidneys compared with the nonischemic kidneys (Figure 6, B and C). This increased methylation corresponds to decreased activity of histone demethylase enzyme (14). Since histone demethylase enzyme requires oxygen for TCA cycle–dependent activation (14), the hypoxic microenvironment during ischemia appears to downregulate its activity. This early epigenetic change may represent the initiation process needed to drive subsequent metabolic rewiring of T cells in post-AKI kidneys. Data from the mouse IRI model exhibited consistent findings, showing higher H3K27Me3 in T cells from ischemic kidneys than those from control mice or sham surgery mice (Figure 6D).

### Glutamine blockade exhibited a protective effect and reduced leukocyte infiltration in ischemic AKI.

Since glutamine utilization is essential for metabolic regulation of effector T cell activation and function, and a therapeutic agent is available that was safe in cancer models, we induced T cell metabolic reprogramming through glutamine antagonism and studied its effect on 2 murine AKI models. Since in vivo use of conventional glutamine antagonists has been hindered by their dose-limiting toxicity (28), we utilized a recently developed prodrug of 6-Diazo-5-oxo-l-norleucine (DON), termed JHU083 (29). JHU083 inhibits a broad range of enzymes involved in glutamine metabolism, including rate-limiting enzyme glutaminase, with improved safety profiles and bioavailability (28).

**Figure 7:**
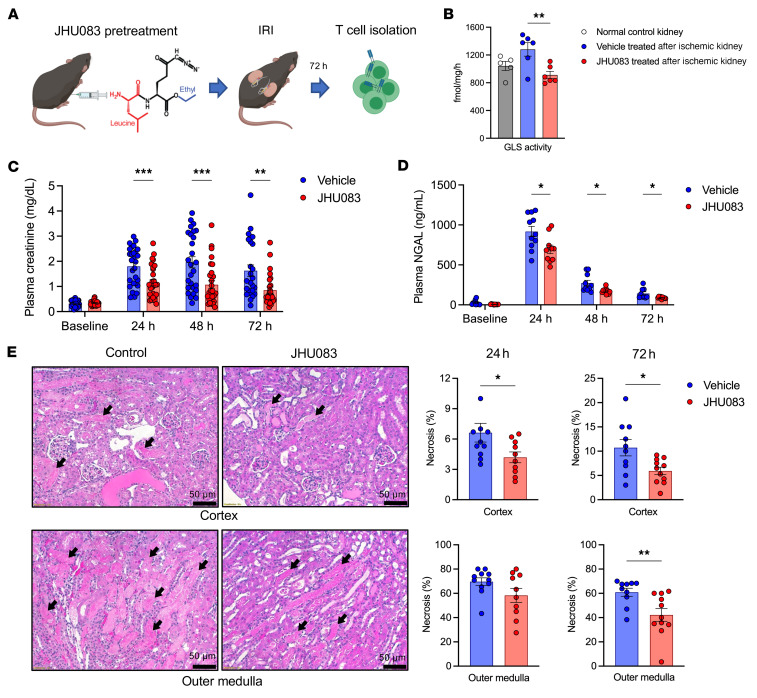
Effect of glutamine blockade in ischemic AKI. (**A**) Schematic of the experimental design. Kidney T cells were isolated 72 hours from ischemia. (**B**) Glutaminase activity in postischemic kidneys was significantly reduced in the JHU083-treated mice compared with the vehicle-treated mice (*n* = 5–6 mice in each group). (**C**) Plasma creatinine concentrations following ischemic AKI. JHU083 treatment significantly improved kidney function. *n* = 36–37 mice in each group. Four mice from the vehicle control group and 2 mice from the JHU083-treated group died on day 2 or 3 following IRI. Data are from 5 independent experiments. (**D**) Plasma NGAL concentrations following ischemic AKI. *n* = 10–11 mice in each group. Data are from 2 independent experiments. (**E**) Histologic findings at 24 hours and 72 hours after ischemic AKI. Necrotic tubules were significantly lower in the JHU083-treated group compared with the vehicle control group. *n* = 10–11 mice in each group; data are from 4 independent experiments. Statistical analyses were performed using 2-tailed *t* test. *P < 0.05; **P < 0.01; ***P < 0.001. AKI, acute kidney injury; GLS, glutaminase; IRI, ischemia/reperfusion injury; NGAL, neutrophil gelatinase-associated lipocalin.

Mice were treated with 1.83 mg/kg of JHU083 or vehicle every other day via intraperitoneal injection and underwent bilateral IRI surgery on day 7 after the initial injection. This dosage regimen has been proven to be tolerable without significant toxicity even with a longer duration of treatment by a previous study (30). Mice were followed up until 72 hours after IRI, and T cells isolated from 72-hour postischemic kidneys were studied (Figure 7A).

To verify that JHU083 suppressed glutamine-related enzymes in kidneys, glutaminase activities were measured in postischemic kidney tissues at 24 hours after IRI. Glutaminase activity showed a trend toward higher levels in vehicle-treated postischemic kidneys (23% increase, *P* = 0.085) compared with the normal kidneys. Treatment with JHU083 significantly reduced glutaminase activities (29% decrease, *P* = 0.008) (Figure 7B).

The JHU083-treated mice showed significantly lower plasma creatinine (at 24 hours, vehicle control versus JHU083, 1.80 ± 0.14 versus 1.15 ± 0.13 mg/dL, *P* = 0.001; at 48 hours, 1.96 ± 0.2 versus 1.07 ± 0.16 mg/dL, *P* = 0.002; at 72 hours, 1.63 ± 0.23 versus 0.85 ± 0.12 mg/dL, *P* = 0.004) and NGAL concentrations (at 24 hours, 916.5 ± 65.2 versus 693.6 ± 53.0 ng/mL, *P* = 0.017; 48 hours, 272.2 ± 34.9 versus 174.5 ± 11.0 ng/mL, *P* = 0.016; 72 hours, 141.6 ± 21.0 versus 88.9 ± 4.1 ng/mL, *P* = 0.025) (Figure 7, C and D) and less injury (cortical necrotic tubules at 24 hours, 6.6% ± 0.9% versus 4.2% ± 0.5%, *P* = 0.039; medullary necrotic tubules at 24 hours, 69.7% ± 3.1% versus 58.3% ± 5.7%, *P* = 0.093; cortical necrotic tubules at 72 hours, 10.7% ± 1.7% versus 5.9% ± 0.8%, *P* = 0.015; medullary necrotic tubules at 72 hours, 60.9% ± 3.4% versus 42.2% ± 5.3%, *P* = 0.009) (Figure 7E).

### Glutamine blockade reduces T cell activation and proliferation in postischemic kidneys.

We analyzed kidney mononuclear cells (KMNCs) in postischemic kidneys to study the effect of JHU083 on T cells. The JHU083 treatment reduced the number of total T cells in postischemic kidneys. The proportions of CD4^+^, CD8^+^, and DN T cells among total T cells (CD45^+^TCRαβ^+^) and regulatory T cells (Tregs) among CD4^+^ T cells were comparable between groups (Supplemental Figure 2).

**Figure 8:**
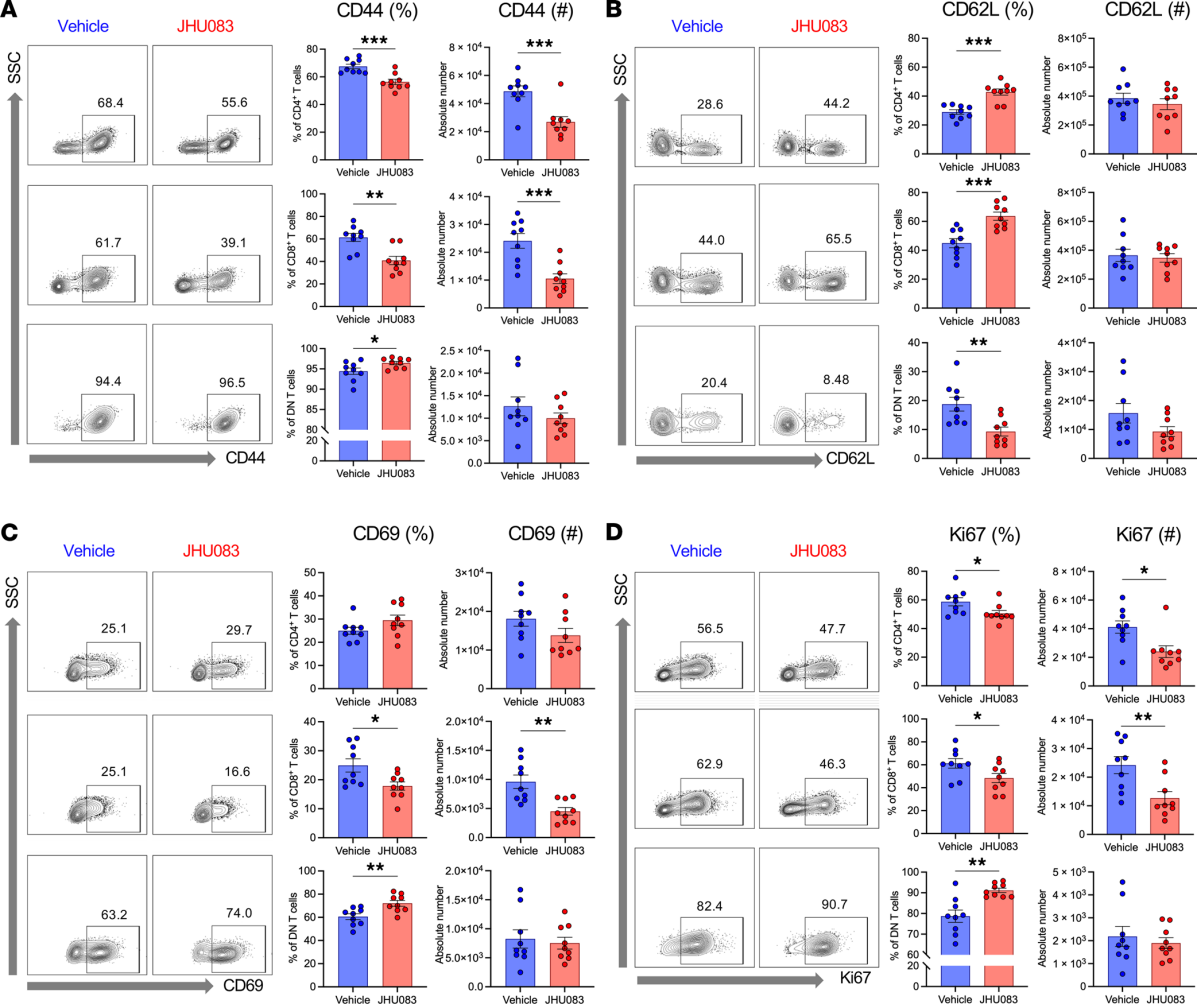
Effects of glutamine blockade on T cell activation and proliferation in postischemic kidneys. (**A** and **B**) JHU083 treatment resulted in significant decrease in CD44 expression and increase in CD62L expression in kidney CD4^+^ and CD8^+^ T cells, suggesting that JHU083-treated postischemic kidneys had fewer effector memory phenotype T cells. (**C**) Activation marker CD69 expression levels on CD8^+^ T cells were reduced in the JHU083-treated group. (**D**) Proliferation marker Ki67 expression in CD4^+^ and CD8^+^ T cells were downregulated in the JHU083-treated group. Kidney T cells were isolated 72 hours from ischemia. Statistical analyses were performed using 2-tailed *t* test (*n* = 9 mice in each group). *P < 0.05; **P < 0.01; ***P < 0.001. DN, double-negative.

Further immunophenotypic characterization of T cells in postischemic kidneys demonstrated that the JHU083 treatment reduced effector and activated phenotypes of CD4^+^ (CD44^+^, 67.5% ± 1.7% versus 56.3% ± 1.9%, *P* < 0.001; CD62L^+^, 29.0% ± 1.6% versus 42.8% ± 2.2%, *P* < 0.001) and CD8^+^ T cells (CD44^+^, 61.4% ± 10.7% versus 40.9% ± 11.2%, *P* = 0.001; CD69^+^, 24.9% ± 2.3% versus 17.8% ± 1.4%, *P* = 0.019; CD62L^+^, 44.9% ± 3.2% versus 63.6% ± 2.8%, *P* < 0.001) (Figure 8). T cell proliferation assessed with Ki67 expression was reduced in CD4^+^ and CD8^+^ T cells from JHU083-treated postischemic kidneys (Ki67^+^, CD4^+^ T cells 58.7% ± 2.9% versus 50.6% ± 2.1%, *P* = 0.035; CD8^+^ T cells 61.2% ± 4.2% versus 48.4% ± 4.1%, *P* = 0.043) (Figure 8). JHU083 treatment did not reduce activation and proliferation of kidney DN T cells, the unconventional subset of kidney αβ T cells, but rather modestly enhanced activation and proliferation (CD44^+^, 94.4% ± 2.4% versus 96.4% ± 1.2%, *P* = 0.038; CD69^+^, 60.6% ± 2.5% versus 72.1% ± 2.5%, *P* = 0.005; Ki67^+^, 78.7% ± 3.0% versus 91.2% ± 1.1%, *P* = 0.001) (Figure 8). We also studied immunophenotypes of splenic T cells from post-IRI mice. Splenic CD4^+^ and CD8^+^ T cells from the JHU083-treated mice showed increased naive phenotypes with low CD44 and high CD62L expression (Supplemental Figure 3). There were changes in metabolic profiles in kidney and splenic T cells after JHU083 treatment (Supplemental Figures 4 and 5).

To test whether this immunologic effect was derived from the glutamine blockade or attenuated kidney injury, we studied normal mice treated with JHU083 or vehicle with the same dosage schedule. We found that CD4^+^ and CD8^+^ T cells in steady-state kidneys were also skewed toward naive phenotypes with lower CD69, CD44, and Ki67 and higher CD62L following glutamine blockade (Supplemental Figure 6). Therefore, changes seen in these markers in postischemic kidneys are likely through JHU083-mediated glutamine blockade.

### Glutamine blockade is not protective against ischemic AKI in T cell–deficient mice.

**Figure 9:**
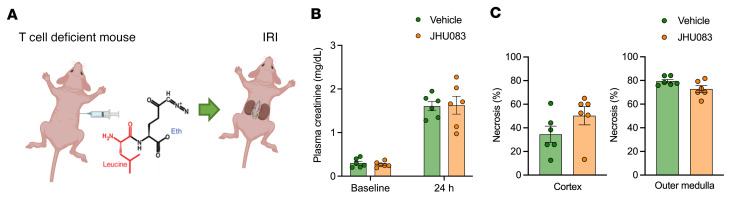
Effect of glutamine blockade in T cell–deficient mice. (**A**) IRI was induced in T cell–deficient mice (*Foxn1*^nu^) with JHU083 pretreatment. (**B** and **C**) JHU083 pretreatment did not alter functional and structural renal outcome. Statistical analyses were performed using 2-tailed *t* test (*n* = 6 mice per each group). AKI, acute kidney injury; IRI, ischemia/reperfusion injury.

To distinguish whether the protective effect of glutamine blockade is by a T cell–dependent mechanism, we induced ischemic AKI and tested the effect of glutamine blockade in T cell–deficient mice (*Foxn1*^nu^) (Figure 9A). We found that glutamine blockade did not confer significant functional (plasma creatinine at 24 hours, vehicle control versus JHU083, 1.61 ± 0.11 mg/dL versus 1.63 ± 0.21 mg/dL, *P* = 0.929) or structural protection (cortical necrotic tubules at 24 hours, 34.6% ± 6.9% versus 50.3% ± 7.7%, *P* = 0.160; medullary necrotic tubules at 24 hours, 79.4% ± 1.5% versus 72.8% ± 2.8%, *P* = 0.067) against ischemic AKI in T cell–deficient mice, unlike the protection observed in WT mice (Figure 9, B and C).

### Glutamine blockade exhibits a protective effect and reduces T cell activation and proliferation in nephrotoxic AKI.

**Figure 10:**
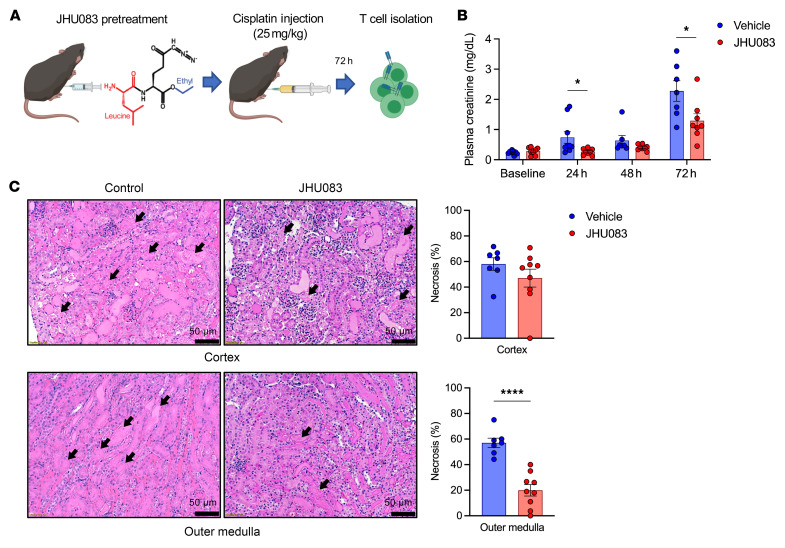
Effect of glutamine blockade in cisplatin-induced AKI. (**A**) Schematic of the experimental design. Kidney T cells were isolated at 72 hours after cisplatin injection. (**B**) Plasma creatinine concentrations following cisplatin-induced AKI. JHU083 treatment significantly attenuated functional renal injury. *n* = 9 mice in each group. Two mice died in the vehicle control group on day 2. (**C**) Histologic findings at 72 hours after cisplatin-induced AKI. Necrotic tubules in outer medulla were significantly lower in the JHU083-treated group. Statistical analyses were performed using 2-tailed *t* test (*n* = 7–9 mice in each group). *P < 0.05; ****P < 0.0001. AKI, acute kidney injury.

Given the significant protective outcomes in ischemic AKI, we subsequently tested the effect of glutamine blockade on a nephrotoxic AKI model, which is another common etiology of clinical AKI. After pretreatment with JHU083 or vehicle, 25 mg/kg of *cis*-diammineplatinum II dichloride (cisplatin) was injected intraperitoneally. T cells were isolated and studied 72 hours after cisplatin treatment (Figure 10A).

Glutamine blockade with JHU083 treatment reduced functional (plasma creatinine at 24 hours, 0.74 ± 0.19 mg/dL versus 0.27 ± 0.04 mg/dL, *P* = 0.039; 48 hours, 0.64 ± 0.16 mg/dL versus 0.41 ± 0.03 mg/dL, *P* = 0.161; 72 hours, 2.28 ± 0.34 mg/dL versus 1.17 ± 0.24 mg/dL, *P* = 0.016) and structural renal injury at 72 hours (cortical necrotic tubules, 58.1% ± 4.9% versus 47.0% ± 7.0%, *P* = 0.243; medullary necrotic tubules, 57.1% ± 3.7% versus 19.9% ± 4.5%, *P* < 0.001) in cisplatin-induced AKI (Figure 10, B and C).

**Figure 11:**
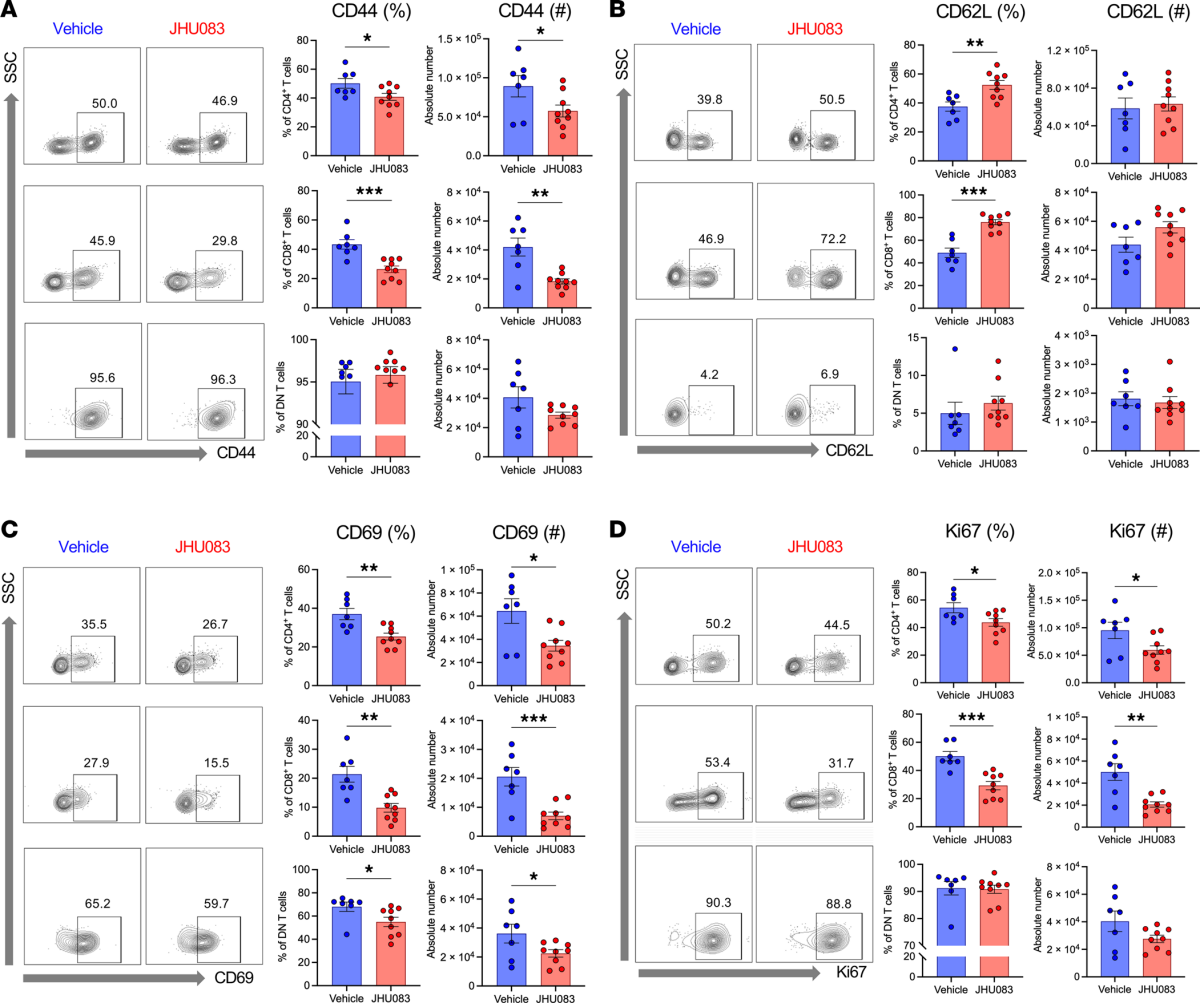
Effects of glutamine blockade on T cell activation and proliferation in cisplatin AKI. (**A** and **B**) JHU083 treatment resulted in significantly reduced CD44 expression and increased CD62L expression in kidney CD4^+^ and CD8^+^ T cells. (**C**) CD69 expression in CD4^+^, CD8^+^, and DN T cells was reduced. (**C**) Percentage of CD62L expression was increased in CD4^+^ and CD8^+^ T cells. (**D**) Ki67 expression in CD4^+^ and CD8^+^ T cells was reduced. Kidney T cells were isolated at 72 hours after cisplatin injection. Statistical analyses were performed using 2-tailed *t* test (*n* = 7–9 mice in each group). *P < 0.05; **P < 0.01; ***P < 0.001. DN, double-negative.

Consistent with immunophenotypic findings in ischemic AKI, JHU083-treated post-cisplatin AKI kidneys had CD4^+^ (CD44^+^, 50.2% ± 3.4% versus 40.9% ± 2.4%, *P* = 0.037; CD62L^+^, 37.5% ± 3.2% versus 52.5% ± 3.0%) and CD8^+^ T cells (CD44^+^, 43.3% ± 3.3% versus 26.4% ± 2.3%, *P* < 0.001; CD62L^+^, 48.9% ± 4.2% versus 76.0% ± 2.3%, *P* < 0.001) with primarily naive phenotypes. JHU083 treatment reduced activation and proliferation of kidney CD4^+^ (CD69^+^, 37.0% ± 2.9% versus 25.4% ± 1.8%, *P* = 0.014; *P* = 0.005; Ki67^+^, 37.0% ± 2.9% versus 25.4% ± 1.8%, *P* = 0.003) and CD8^+^ T cells (CD69^+^, 21.4% ± 2.7% versus 9.8% ± 1.5%, *P* = 0.001; Ki67^+^, 21.4% ± 2.7% versus 9.8% ± 1.5%, *P* = 0.001) (Figure 11).

### Glutamine blockade inhibited T cell activation and proliferation under hypoxia.

**Figure 12:**
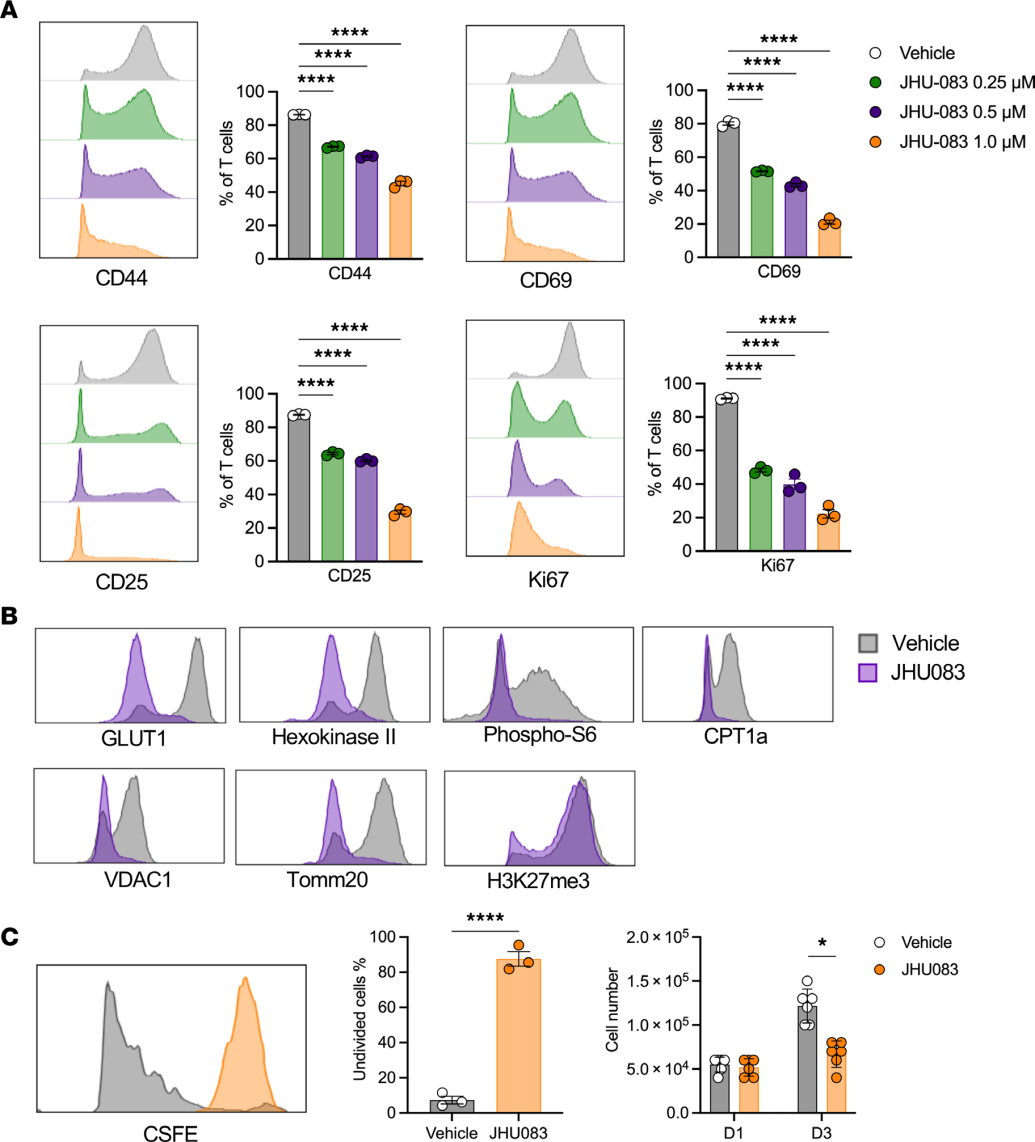
Effects of glutamine blockade on T cell activation and proliferation in vitro hypoxia. CD3/CD28-stimulated T cells were treated with JHU083 or vehicle and underwent 24-hour hypoxia followed by reoxygenation. (**A**) JHU083 treatment reduced CD44, CD69, CD25, and Ki67 expression in a dose-dependent manner. Statistical analyses were performed using 1-way ANOVA followed by Tukey’s post hoc analysis. (**B**) Histograms comparing vehicle-treated T cells and JHU083-treated T cells. (**C**) CD3/CD28-stimulated kidney T cells were exposed to hypoxia followed by reoxygenation. JHU083 treatment reduced kidney T cell proliferation significantly. Statistical analyses were performed using 2-tailed *t* test. *P < 0.05; ****P < 0.0001. DN, double-negative.

In vitro studies were performed to evaluate the effect of glutamine blockade on activated T cells under hypoxia followed by reoxygenation. Splenic T cells (CD45^+^TCRαβ^+^) were isolated and cultured using media containing 0.25 μM, 0.5 μM, or 1 μM of JHU083 or vehicle. After 24 hours from the culture under CD3/CD28 stimulation, cells were exposed to hypoxia for 24 hours followed by reoxygenation. While the majority of vehicle-treated T cells expressed CD44, CD69, CD25, and Ki67, indicating activation and proliferation induced by CD3/CD28, JHU083-treated T cells showed reduced expression of activation and proliferation markers compared with the vehicle-treated cells in a dose-dependent manner (Figure 12A). Metabolic profiles of JHU083-treated T cells exhibited unstimulated T cell phenotypes, suggesting that glutamine blockade inhibits CD3/CD28-mediated T cell activation in vitro (Figure 12B).

Glutamine blocking effect on the kidney T cell proliferation was assessed with CFSE analysis. FACS-sorted kidney T cells (CD45^+^TCRαβ^+^) were cultured with media containing 1 μM JHU083 or vehicle and underwent hypoxia followed by reoxygenation. There was a significantly higher proportion of undivided cells in the JHU083-treated cells (*P* < 0.001). The cell numbers were significantly lower on day 3 (Figure 12C).

## Discussion

Unlike traditional concepts of adaptive immunity as relatively late responders during inflammation, it is now well established that T cells traffic into kidneys at a very early point after AKI and regulate early injury responses (18, 19). There has been a large body of work in both kidneys and other solid organs demonstrating an important role for T cells in acute tissue injury (31–34). Given these roles for T cells in AKI and emerging data on importance of T cell metabolism for their function, we hypothesized that T cells undergo metabolic reprogramming during AKI and modulation of T cell metabolism could modify AKI outcome. We first performed a descriptive survey with spectral flow cytometry and used machine learning programs to demonstrate the metabolic changes in kidney and splenic T cells after mouse AKI. We identified a distinct T cell subset, likely unique to post-AKI kidneys, exhibiting reduced mTOR activity and OXPHOS and fatty acid oxidation machineries but maintaining glycolysis. Furthermore, an early T cell epigenetic modification and increased glutaminase activity were found during ischemia. We then studied human kidney to demonstrate clinical relevance of the mouse findings. We then embarked on therapeutic and mechanistic studies targeting T cell metabolism with the glutamine antagonist JHU083. JHU083 was protective in both ischemic and nephrotoxic models of AKI and changed kidney CD4^+^ and CD8^+^ T cells toward naive phenotypes. In vitro studies demonstrated that hypoxia induced upregulation of glycolysis in kidney T cells, and glutamine blockade reduced their proliferation.

Spectral flow cytometry overcomes limited multiplexing capacity of conventional flow cytometry by allowing highly complex multicolor panels (35). We also used an unsupervised machine learning algorithm to discover metabolic reprogramming of T cells. This computational approach provides data visualization and facilitates identification of unexpected cells or previously undefined cell populations for downstream analyses (35). Given the recent introduction of spectral flow cytometers, multidimensional computational analysis is becoming increasingly used in immunology research. We predicted that combining metabolic markers with computational analyses beyond the conventional immunophenotypic markers would provide a deeper understanding of kidney immune cells across the various types of kidney disease.

We found that the metabolic reprogramming of T cells occurred during the very early stage of AKI within 4 hours of reperfusion. We demonstrated that expression of histone methylation marker on T cells, which is affected by TCA cycle metabolites as well as many other cellular pathways, was increased during ischemia. This epigenetic modification drives T cell metabolic reprogramming, and the bidirectional relationship between epigenetic imprinting and T cell metabolism is a recently emerging area (36). Importantly, we found that T cells maintained glycolysis machinery, whereas OXPHOS- and fatty acid oxidation–related enzymes were downregulated. VDAC1 is the most prevalent protein in the mitochondrial outer membranes and functions as a major transporter of metabolites (37). However, these cells maintained Tomm20, another OXPHOS-related protein. This discrepancy between expected Tomm20 and VDAC1 expression likely represents nuances in the response to kidney IRI. Since mTOR activity is known to be inhibited under cellular hypoxia and lack of nutrients (38), low mTOR activity may represent the consequence of hypoxic stress, leading to a decrease in biosynthesis. Functional relevance of these metabolic changes needs to addressed in the future.

In contrast to kidney T cells during AKI, splenic T cells did not undergo hypoxia followed by reperfusion or direct antigen exposure during ischemic AKI. However, splenic T cells still exhibited metabolic reprogramming following experimental AKI. Metabolic machineries were globally upregulated in post-AKI spleens, resembling metabolic signatures of activated T cells by viral infection. This finding is in line with previous studies demonstrating extrarenal T cell activation in AKI (39, 40). Accumulating evidence suggests that this distant immunologic effect of AKI is associated with multiorgan dysfunction in AKI, affecting patients’ overall outcome and mortality (27). Metabolic reprograming of extrarenal T cells followed by AKI from our data highlights T cell–mediated distant organ crosstalk in AKI, emphasizing its importance as a potential therapeutic target. The underlying mechanism that induces splenic T cell metabolic reprogramming in AKI needs to be further explored.

Our in vitro data demonstrated that hypoxia per se was capable of inducing metabolic rewiring of kidney T cells with increased glycolysis. This upregulation of glycolysis following hypoxia exposure has been demonstrated by a previous study using T cells from lymphoid organs (41). However, the metabolic landscape of kidney T cells under in vitro hypoxia was different from the in vivo findings that showed more complex signatures. One possible reason for this difference is cell culture media contain much higher concentrations of nutrients than those present in the harsh metabolic environment within tissue (42).

T cell activation is a glutamine-dependent process, and TCR stimulation signal mediates glutamine uptake in naive T cells (43, 44). Activated inflammatory T cells show 5- to 10-fold increase in glutamine uptake. Other amino acids are unable to replace glutamine because transport capacity of other amino acids on T cells is insufficient to compensate glutamine utilization (44). Therefore, based on exceptionally high glutamine demands of the effector T cells, we induced metabolic reprogramming with glutamine blockade. Achieving in vivo glutamine antagonism was previously limited because of dose-limiting gastrointestinal toxicity of conventional glutamine antagonist DON (28). A prodrug strategy was used to change pharmacokinetic profiles and reduce toxicity (45). We therefore utilized the recently developed prodrug form of DON, JHU083, designed to increase biosafety and inhibit a broad range of glutamine-requiring reactions (28). We demonstrated that glutamine antagonism markedly improved functional and structural renal outcomes in both ischemic and nephrotoxic models of AKI. JHU083 prevented kidney CD4^+^ and CD8^+^ T cell activation, steering these cells to a naive-like phenotype with low proliferative capacity. Moreover, T cell–deficient mice were not protected by JHU083 treatment. Thus, the mechanism by which glutamine antagonism has a protective effect in experimental AKI could be attributed to inhibiting effector functions of CD4^+^ T cells. Our finding is in accordance with the previous in vitro study that demonstrated amelioration of glutamine-dependent T cell activation by using glutaminase inhibitors (46). However, we cannot rule out important effects on other immune or non-immune cells such as renal tubular epithelial cells.

Targeting metabolism to regulate immune response is a rapidly evolving area (14). Our findings are consistent with previous studies, which have shown beneficial effects of glutamine antagonism in infections, autoimmune diseases, and alloimmunity models (22, 47–49). Given that we found that glutamine antagonism provided a protective effect on cisplatin-induced AKI, and the published antitumor effect of glutamine antagonism (20), combining glutamine blocking strategy with cisplatin-based chemotherapy regimens could be a promising approach to enhance antineoplastic activity of cisplatin while providing renoprotection.

Despite the low selectivity of drugs that target metabolism, cellular selectivity can be achieved through cellular metabolic demands and programs (22). While ordinary cells have more flexible metabolism, effector CD4^+^ and CD8^+^ T cells have the greatest demand for glutaminolysis. Thus, glutamine antagonism can selectively inhibit those cells, not altering normal cellular homeostatic function. We found that DN T cells, a recently emerging kidney T cell subset that has shown a protective effect on AKI (50–53), were resistant to glutamine blockade, maintaining their activation marker expression and high proliferation capacity. This suggests that DN T cells rely on other metabolic machinery rather than glutaminolysis, a topic of further investigation. Although it is known that tubular epithelial cells are also one of the most energy-demanding cells during kidney injury, they rely on fatty acid oxidation as a major fuel source, unlike effector T cells (54, 55).

Although strategies targeting immune reconstitution to suppress T cell–mediated inflammation in AKI have been tested previously with conventional immunosuppressive drugs, mTOR inhibitor or mycophenolate mofetil, they failed to show a protective effect (56, 57). The negative outcome was associated with inadvertent suppression of regulatory cell subsets that have a protective role in AKI (56, 57). Given this finding, instead of suppressing whole immunity, selective suppression of pathogenic effector T cell subsets appears to be important to achieve a protective effect. Therefore, targeting a metabolic pathway that is selectively required for pathogenic T cells might be a useful strategy to improve AKI outcomes.

However, glutamine supplementation has been shown to have a contrasting protective role in some studies (58–60). A recent study proposed modulation of tubular cell apoptosis signaling and OXPHOS as probable mechanisms of glutamine-mediated protective effect (60). A large multicenter clinical trial demonstrated that parenteral glutamine supplementation was associated with increased mortality and hospital stay, especially in patients with renal dysfunction, but the mechanisms remain uncertain (61, 62). A few studies that addressed immunologic effects showed that glutamine supplementation was associated with enhanced Th1 polarization, reduced circulating Tregs, and increased renal IL-1 and IL-6 expression (63, 64). Future studies will be required to understand the underlying mechanism of negative clinical outcomes associated with glutamine supplementation.

The current study has several limitations. First, although we focused on T cells, which play important roles in AKI pathogenesis, other types of immune cells in kidneys are also involved in AKI pathogenesis (4). Therefore, future studies addressing metabolism of non–T cell populations such as neutrophils, mononuclear phagocytic cells, B cells, or innate lymphoid cells are warranted. Thus, we cannot rule out a collateral effect of JHU083 on other types of immune cells in kidneys, or even non-immune epithelial cells. Second, to achieve T cell metabolic reprogramming, we pharmacologically targeted glutamine-related pathways because of a key role in T cell metabolism and availability of JHU083 to us, as well as its safety in cancer models, but there are metabolic inhibitors that target other metabolic pathways (65). Further studies using different inhibitors targeting non-glutamine pathways or combinations with glutamine blockade will provide a deeper understanding of T cell metabolism in AKI. Since T cells play important roles in renal recovery and repair as well as early injury (31), suppressing the T cell–mediated inflammatory process may inadvertently disrupt the regeneration and recovery process (66). Since our study focused on the early injury phase and local tissue damage, effects of glutamine blockade on long-term renal effects and distant organ effects need to be investigated. Another limitation was that we did not directly measure cellular metabolites but measured enzymes and receptors involved in metabolic pathways. Given that enzyme levels may not necessarily reflect cellular metabolism, measuring cellular metabolites directly using biochemical approaches such as mass spectrometry can provide detailed information for each metabolic pathway (67). However, a caveat of this technique is that this measure provides average metabolomics of whole tissue, not specific cellular insight. Given the uniqueness of T cell metabolic programming (14), metabolic profiles of whole kidney extracts may differ from T cell metabolic profiles. It is important to note that renal ammoniagenesis is dependent on glutamine metabolism, although this was not evaluated in our study. This suggests that glutamine antagonism may have altered renal acid-base homeostasis by ameliorating renal ammoniagenesis, which needs to be investigated by future studies. It should also be noted that human data were limited to preischemic and ischemic nephrectomized kidneys because there was no reperfusion in nephrectomy samples like the mouse IRI model. Future studies will also need to be performed going into more depth on the precise mechanisms of JHU083 effects on kidney T cell populations.

Despite these and other limitations, our study has important future scientific and clinical implications. We demonstrated the immune-metabolic phenotyping changes of kidney T cells in mice and humans at baseline and during AKI. This approach has implications for non-AKI diseases of the kidney ranging from glomerulonephritis to allograft rejection. Furthermore, our findings are relevant for acute ischemic and toxic injury to other organs like liver, heart, and brain. We also demonstrated that pharmacologic intervention targeting glutamine in T cell metabolism can improve both ischemic and nephrotoxic AKI outcomes, with human translational potential.

## Methods

### Mice.

Seven-week-old male C57BL/6J WT mice and T cell–deficient mice (*Foxn1*^nu^ homozygous) were purchased from The Jackson Laboratory and bred under specific pathogen–free conditions at the Johns Hopkins University animal facility. C57BL/6J WT mice were infected with LCMV Armstrong virus (2 × 10^5^ PFU/mouse, i.p.) provided by Susan Kaech (Salk Institute, La Jolla, California, USA). Organs were collected on day 7 at peak of acute infection.

### Ischemic AKI model.

Mice were anesthetized with an i.p. injection of pentobarbital (75 mg/kg; Akorn). After shaving of abdominal hair, mice were placed onto a thermostatically controlled heating table. Abdominal midline incision was performed, and both renal pedicles were dissected and clamped for 30 minutes for WT mice and 45 minutes for T cell–deficient mice (to achieve similar injury responses given protection by T cell deficiency in these mice) with a microvascular clamp (Roboz Surgical Instrument Co). After 30 or 45 minutes, microvascular clamps were released from renal pedicles, and the kidneys were inspected to confirm reperfusion. During the surgery, mice were kept well hydrated with 1 mL of warm sterile 0.9% saline. After being sutured, mice were allowed to recover with free access to chow and water. Sham surgeries were performed identically without clamping of renal pedicles.

### Cisplatin AKI model.

Cisplatin (MilliporeSigma) was freshly dissolved into 0.9% saline (1 mg/mL) on the day of injection. The dissolved solution was incubated in water bath at 40°C for 10 minutes to achieve complete dissolution. A single 25 mg/kg dose of cisplatin was injected i.p.

### Assessment of kidney function.

Blood samples were obtained at 0, 24, 48, and 72 hours after IRI or cisplatin injection through tail vein collection. Plasma creatinine concentration was measured by Cobas Mira Plus automated analyzer system (Roche) with creatinine reagent (Pointe Scientific Inc). Plasma NGAL was measured by mouse lipocalin-2/NGAL quantikine ELISA kits (R&D Systems).

### Tissue histological analysis.

At 24 hours after IRI and 72 hours after cisplatin injection, mice were anesthetized with ketamine (130 mg/kg; VetOne) and xylazine (7 mg/kg; Akorn) i.p. injection. After exsanguination, kidneys were collected and fixed with 10% buffered formalin followed by paraffin embedding. Tissue sections were stained with hematoxylin and eosin. A renal pathologist scored necrotic tubules in a blinded fashion.

### Isolation of KMNCs and splenocytes.

KMNCs were isolated according to our previously described Percoll density gradient protocol (18). Briefly, decapsulated kidneys were immersed and incubated in collagenase D (2 mg/mL; MilliporeSigma) solution for 30 minutes at 37°C. Samples were strained through 70 μm cell strainer (BD Biosciences), washed, and resuspended in 40% Percoll (GE Healthcare), followed by gentle overlaying onto 80% Percoll. After centrifugation at 1,500*g* for 30 minutes in brake-off mode at room temperature, KMNCs were collected from interface between 40% and 80% Percoll. Spleens were strained through 40 μm cell strainer (BD Biosciences) and incubated with ammonium-chloride-potassium lysis buffer (Quality Biological) for 3 minutes. Collected cells were washed and resuspended with Roswell Park Memorial Institute (RPMI) 1640 media (Thermo Fisher Scientific) containing 5% fetal bovine serum (FBS, Thermo Fisher Scientific). Cells were counted on hemocytometer using trypan blue exclusion under a microscope (IMT-2, Olympus).

### Isolation of human KMNCs.

Human kidney tissues were procured from normal portions of renal cell carcinoma nephrectomy kidneys from individuals recruited by Johns Hopkins University. Preischemic kidney tissues were obtained before clamping of renal hilum, whereas ischemic kidney tissues were obtained after clamping of renal hilum. Ischemia time at body temperature for ischemic kidney tissues was 35 to 45 minutes. The obtained kidney tissues were immediately kept on ice and digested according to the above-described protocol. KMNCs were viably cryopreserved in FBS with 10% DMSO (Thermo Fisher Scientific) for downstream analyses.

### Spectral flow cytometry.

Cells were washed once with phosphate-buffered saline (PBS) and stained with viability dye Zombie NIR Fixable Viability (BioLegend) for 15 minutes at room temperature. After washing with Cell Staining Buffer (BioLegend), cells were preincubated with anti-CD16/CD32 Fc receptor blocking antibody (clone S17011E, BioLegend, 156604) for 15 minutes to prevent nonspecific antibody binding. Subsequently, surface staining was performed in 50 μL of BD Horizon Brilliant Stain buffer and surface staining antibody cocktail for 30 minutes at 4°C: Pacific Blue anti-CD44 (clone IM7, BioLegend, 103020), BV510 anti-CD8 (clone 53-6.7, BioLegend, 100752), BV570 anti-CD45 (clone 30-F11, BioLegend, 103136), BV605 anti-CD69 (clone H1.2F3, BioLegend, 104530), BV650 anti-NK1.1 (clone PK136, BioLegend, 108736), BV711 anti-PD1 (clone 29F.1A12, BioLegend, 135231), BV785 anti-TCRβ (clone H57-597, BioLegend, 109249), PE-Cy5.5 anti-CD25 (clone PC61.5, Thermo Fisher Scientific, 35-0251-82), APC-R700 anti-CD62L (clone MEL-14, BD Biosciences, 565159), and APC-Fire810 anti-CD4 (clone GK1.5, BioLegend, 100480). Cells were fixed and permeabilized with Foxp3/transcription factor staining kit (Thermo Fisher Scientific) for 30 minutes at room temperature and washed with permeabilization/wash buffer (Thermo Fisher Scientific). Intracellular staining was conducted in 50 μL of permeabilization/wash buffer with intracellular staining antibody cocktail for 45 minutes at room temperature: BV421 anti-Ki67 (clone 16A8, BioLegend, 652411), Alexa Fluor 488 anti-CPT1a (clone 8F6AE9, Abcam, ab171449), Alexa Fluor 532 anti-VDAC1 (clone 20B12AF2, Abcam, ab14734), PerCP-efluor 710 anti-FoxP3 (clone FJK-16S, Thermo Fisher Scientific, 46-5773-82), PE anti-GLUT1 (clone EPR3915, Abcam, ab209449), Alexa Fluor 594 anti–p-S6 (clone D68F8, Cell Signaling Technology, 9468), PE-Cy5 anti-HKII (clone EPR20839, Abcam, ab228819), PE-Cy7 anti-H3K27me3 (clone C36B11, Cell Signaling Technology, 91611), and Alexa Fluor 647 anti-Tomm20 (clone EPR15581-54, Abcam, ab209606). For glutaminase measurement, intracellular staining was conducted with antibody PE-Cy5 anti-glutaminase (GeneTex, GTX81012). After staining, cells were washed with permeabilization/wash buffer, then resuspended in Cell Staining Buffer. Samples were analyzed by 4-laser Aurora spectral flow cytometer (Cytek).

For the human kidney, cryopreserved KMNCs were thawed and washed with PBS. Cells were stained for viability assay with Zombie NIR for 15 minutes at room temperature. After preincubation with anti-CD16/CD32, cells were stained as described above with the following fluorochrome-labeled antibodies for surface and intracellular staining: BV786 anti-CD3 (clone SK7, BD Biosciences, 563800), BV480 anti-CD8 (clone RPA-T8, BD Biosciences, 566121), BV570 anti-CD45RA (clone HI100, BioLegend, 304132), BV650 anti-CCR7 (clone G043H7, BioLegend, 353234), BV510 anti-CD25 (clone M-A251, BD Biosciences, 563352), BV711 anti-PD1 (clone EH12.2H7, BioLegend, 329928), PE-Cy5 anti-CD4 (clone OKT4, BioLegend, 317412), PE-Cy5.5 anti-CD69 (clone CH/4, Thermo Fisher Scientific, MHCD6918), APC anti-CD49a (clone TS2/7, BioLegend, 328314), APC-Cy7 anti-CD19 (clone SJ25C1, BioLegend, 363006), APC-Cy7 anti-CD56 (clone 5.1H11, BioLegend, 362512), Pacific Blue anti-FoxP3 (clone 206D, BioLegend, 320116), Alexa Fluor 405 anti-Tomm20 (EPR15581-54, Abcam, ab210047), AF532 anti-VDAC1 (clone 20B12AF2, Abcam, ab14734), Alexa Fluor 488 anti-CPT1a (clone 8F6AE9, Abcam, ab171449), PE H3K27me3 (clone C36B11, Cell Signaling Technology, 40724), Alexa Fluor 680 anti-HKII (clone EPR20839, Abcam, ab228819), and Alexa Fluor 647 GLUT1 (clone EPR3915, Abcam, ab195020).

### High-dimensional flow cytometry data analysis.

The acquired raw data from the spectral flow cytometer were unmixed by SpectroFlo software (Cytek). Unmixed data were first curated with FlowJo 10.8 software (BD Biosciences) to remove debris, doublets, and dead cells. Curated data were downsampled and concatenated to conduct downstream analyses. High-dimensional unbiased analyses were performed using FlowJo plugin UMAP 3.1.

### In vivo glutamine blockade.

Glutamine antagonist JHU083 was synthesized as previously described (29) and dissolved in 50 mM 10 μM HEPES-buffered 0.9% saline. Aliquot stocks were stored at –80°C and thawed right before injection. Before the IRI surgery or cisplatin injection, mice were administered 1.83 mg/kg JHU083 (equivalent to 1 mg/kg DON) or vehicle (50 mM HEPES-buffered 0.9% saline) every other day via i.p. injection. Following 4 consecutive dosages, mice underwent IRI surgery or cisplatin injection. The last additional dose was given at 24 hours after IRI or cisplatin injection. This dosage regimen was chosen based on efficiency and toxicity data from the previous study (30). Body weight was monitored every other day, and no significant differences between groups were observed due to JHU083 treatment.

### Kidney and splenic T cell isolation for cell culture.

Kidney T cells were isolated using FACS. Briefly, single-cell suspension of KMNCs was preincubated with anti-CD16/CD32 Fc block (clone S17011E, BioLegend, 156604) stained in Cell Staining Buffer (BioLegend) with fluorochrome-labeled antibodies: APC-Cy7 anti-CD45 (clone 30-F11, BioLegend, 103116) and BV421 anti-TCRβ (clone H57-957, BioLegend, 109230). Live Dead Aqua (Thermo Fisher Scientific) was stained for viability assay. Live Dead Aqua^–^CD45^+^TCRβ^+^ cells were sorted with FACSAria II Cell Sorter (BD Biosciences). Splenic T cells were isolated from single-cell suspension of spleens using a T Cell Isolation Kit II (Miltenyi Biotec) according to the manufacturer’s guidelines.

### In vitro T cell culture.

We coated 48-well, flat-bottom plates with 5 μg/mL anti-CD3e (clone 145-2C11, Tonbo Biosciences, 40-0031-M001) in PBS and incubated at 4°C overnight. Anti-CD3e–coated plates were washed with PBS, and isolated cells were cultured in RPMI 1640 GlutaMAX (Thermo Fisher Scientific) supplemented with 10% FBS (Thermo Fisher Scientific), 10 μM HEPES buffer (Thermo Fisher Scientific), 100 μM non-essential amino acid solution (MilliporeSigma), and 55 μM 2-mercaptoethanol (MilliporeSigma) with/without JHU083. After 72 hours of culture, cells were stained with flow cytometry antibodies as previously described.

### In vitro hypoxia induction.

Cultured cells were incubated under hypoxic (1% O_2_) condition according to the following protocol. For hypoxia induction, culture plates were placed in a modular incubator chamber, and the chamber was flushed with gas mixture containing 1% O_2_, 5% CO_2_, and 94% N_2_ for 3 minutes. After flushing, the incubator was completely sealed and placed into cell culture incubator for 24 hours.

### Glutaminase activity analysis.

Glutaminase activity measurements in kidney samples were adapted from previously described protocols (68). Briefly, kidneys were homogenized using Kimble Biomasher II and then sonicated (3 pulses of 15-second duration on ice using Kontes Micro Ultrasonic Cell Disrupter) in ice-cold potassium phosphate buffer (45 mM, pH 8.2) containing protease inhibitors (Roche, Complete Protease Inhibitor Cocktail, 1 tablet in 10 mL) and incubated with [^3^H]-glutamine (0.04 μM, 0.91 μCi) for 90 minutes at room temperature. The reactions were carried out in 50 μL reaction volumes in a 96-well microplate. At the end of the reaction period, the assay was terminated upon the addition of imidazole buffer (20 mM, pH 7). We used 96-well spin columns packed with strong anion ion-exchange resin (Bio-Rad, AG 1-X2 Resin, 200-400 mesh, chloride form) to separate the substrate and the reaction product. Unreacted [^3^H]-glutamine was removed by washing with imidazole buffer. [^3^H]-glutamate, the reaction product, was then eluted with 0.1N HCl and analyzed for radioactivity using PerkinElmer’s TopCount instrument in conjunction with the company’s 96-well LumaPlates. Finally, total protein measurements were carried as per manufacturer’s instructions using BioRad’s Detergent Compatible Protein Assay kit and data are presented as fmol/mg/h.

### Statistics.

Data were expressed as mean ± SEM. Two group means were compared with 2-tailed *t* test. Three or more group means were compared using 1-way or 2-way ANOVA followed by Tukey’s post hoc analyses. All statistical tests were performed using Prism version 9 (GraphPad Software). *P* values less than 0.05 were considered statistically significant.

### Study approval.

The human study was performed in accordance with the Declaration of Helsinki and approved by the Johns Hopkins Medicine Institutional Review Board (CR00031498). Written informed consent was received prior to participation, and all data were deidentified. The animal studies were conducted according to the Johns Hopkins University Institutional Animal Care and Use Committee–approved protocols.

### Data availability.

The data that support the findings of this study are available on request from the corresponding author.

## Author contributions

HR designed the study. KL and HR drafted the manuscript. KL, EAL, SG, CHP, JTK, and SN performed the experiments. KL, EAT, and HR analyzed and interpreted the data. PMP collected and provided human kidney samples. LJA analyzed histology data. BSS developed and provided JHU083. AGT conducted the glutaminase activity assays. EAT, SG, CHP, JTK, SN, PMP, LJA, AGT, and BSS revised the manuscript. All authors approved the final version of the manuscript.

## Supplementary Material

Supplemental data

## Version 1 05/11/2023

In-Press Preview

## Version 2 06/22/2023

Electronic publication
