# Supplementary material for: T cell metabolic reprogramming in acute kidney injury and protection by glutamine blockade
Source: JCI Insight. 2023 Jun 22;8(12):e160345. doi: 10.1172/jci.insight.160345 (PMC10371253; doi:10.1172/jci.insight.160345)
Supplement: Supplemental data [file jciinsight-8-160345-s043.pdf]

Supplemental Figure 1

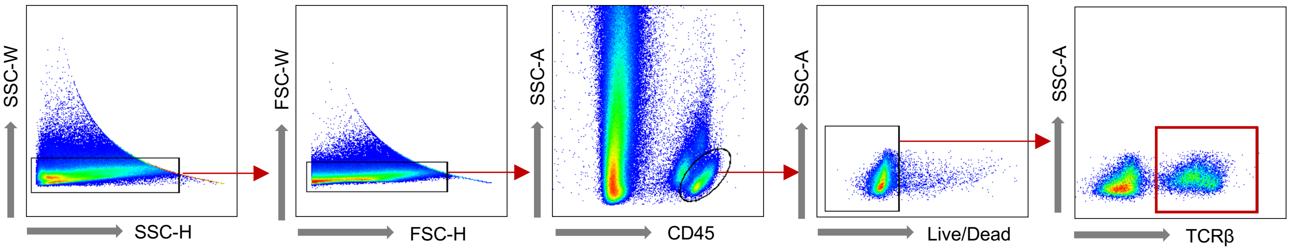

Supplemental Figure 1. Gating strategy for kidney T cells. Isolated KMNCs were analyzed with spectral flow cytometer as described in Methods. After single cell gating, lymphocytes were identified with SSC-A and $CD45^{high}$ gating. αβ T cells were identified from live lymphocytes population with $TCR\beta^{+}$ gating.

KMNCs, kidney mononuclear cells

Supplemental Figure 2

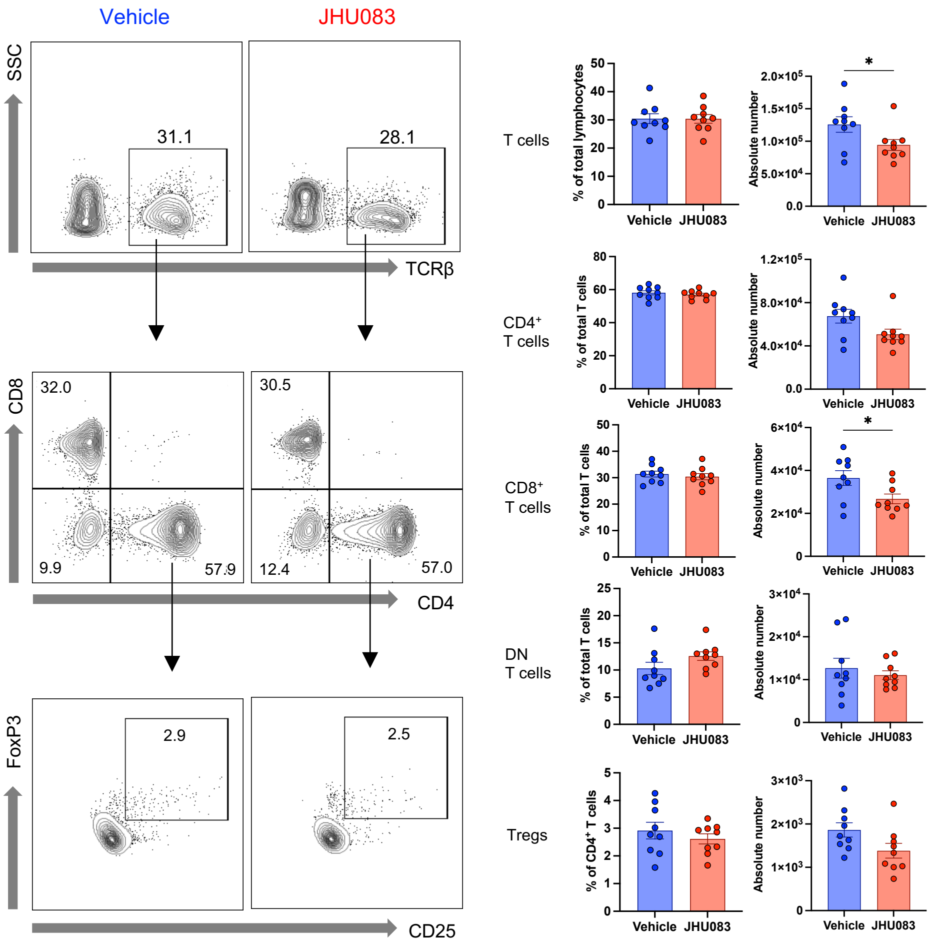

Supplemental Figure 2. Effects of glutamine blockade on kidney T cell populations in post-ischemic kidneys. Post-ischemic kidneys from the JHU083 treated mice had a reduced number of total T cells. Proportions of $CD4^+$, $CD8^+$, and DN T cells among total T cells were comparable between groups. Percentage of Tregs among $CD4^+$ T cells was comparable. Statistical analyses were performed using two-tailed *t* test ($n = 9$ mice in each group). $^*P < 0.05$; $^{**}P < 0.01$; $^{***}P < 0.001$.
DN, double-negative.

Supplemental Figure 3

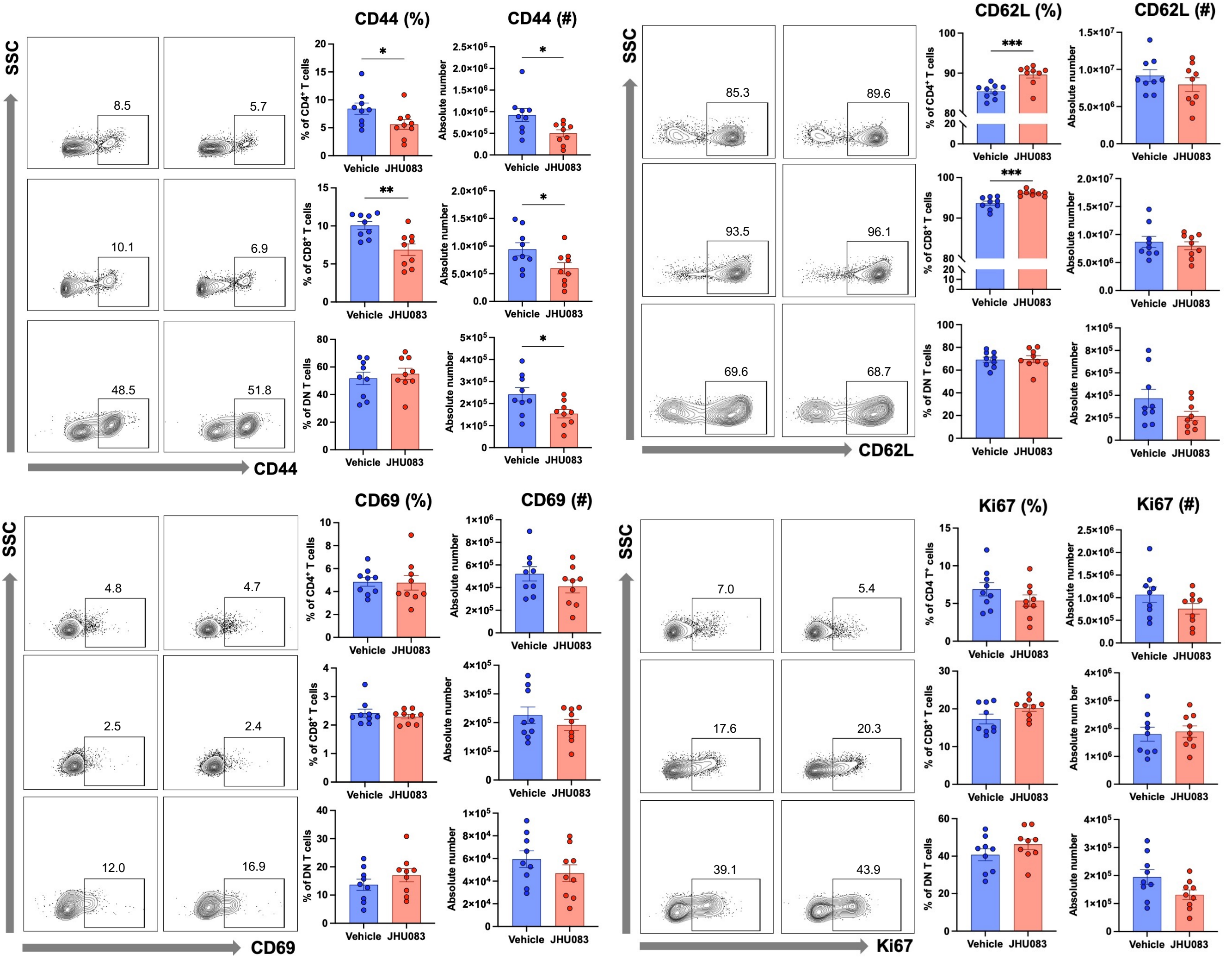

Supplemental Figure 3. Effects of glutamine blockade on splenic T cell phenotypes in ischemic AKI. JHU083 treatment reduced CD44 expression and increased CD62L expression in splenic $CD4^+$ and CD8 T^+ cells from post-IRI mice. . Statistical analyses were performed using two-tailed *t* test (*n* = 9 mice in each group). $^*P < 0.05$; $^{**}P < 0.01$; $^{***}P < 0.001$

DN, double-negative.

Supplemental Figure 4

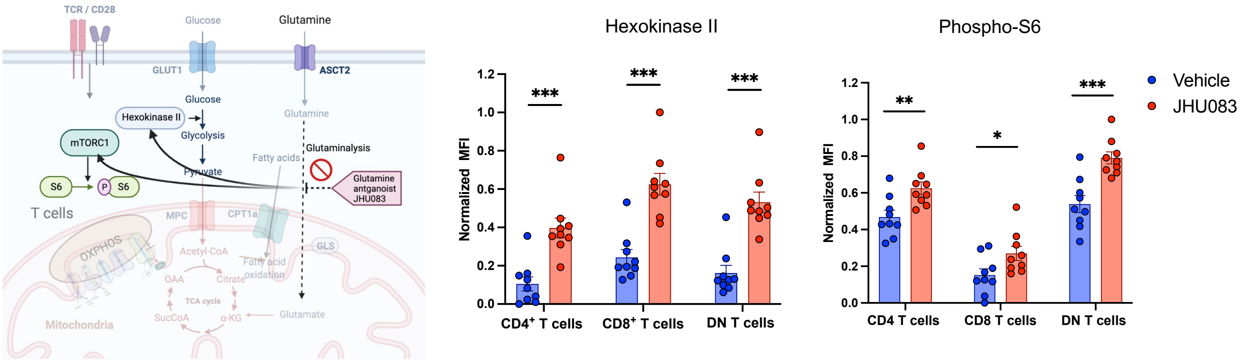

Figure 8. Effect of glutamine blockade on kidney T cell metabolism in ischemic AKI. JHU083 treatment upregulated hexokinase II and pS6 in T cells from post-ischemic kidneys. Statistical analyses were performed using two-tailed *t* test ($n = 9$ mice in each group). *$P < 0.05$; **$P < 0.01$; ***$P < 0.001$.

DN, double-negative; GLS, glutaminase; pS6, phospho-S6 ribosomal protein.

Supplemental Figure 5

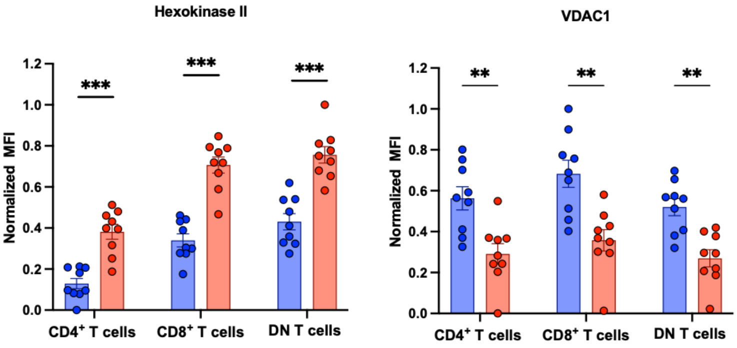

Supplemental Figure 5. Effect of glutamine blockade on splenic T cell metabolism in ischemic AKI. JHU083 treatment upregulated hexokinase II and downregulated VDAC1 in post-AKI splenic T cells. Statistical analyses were performed using two-tailed *t* test ($n = 9$ mice in each group). *$P < 0.05$; **$P < 0.01$; ***$P < 0.001$.

DN, double-negative; VDAC1, voltage-dependent anion channel 1.

Supplemental Figure 6

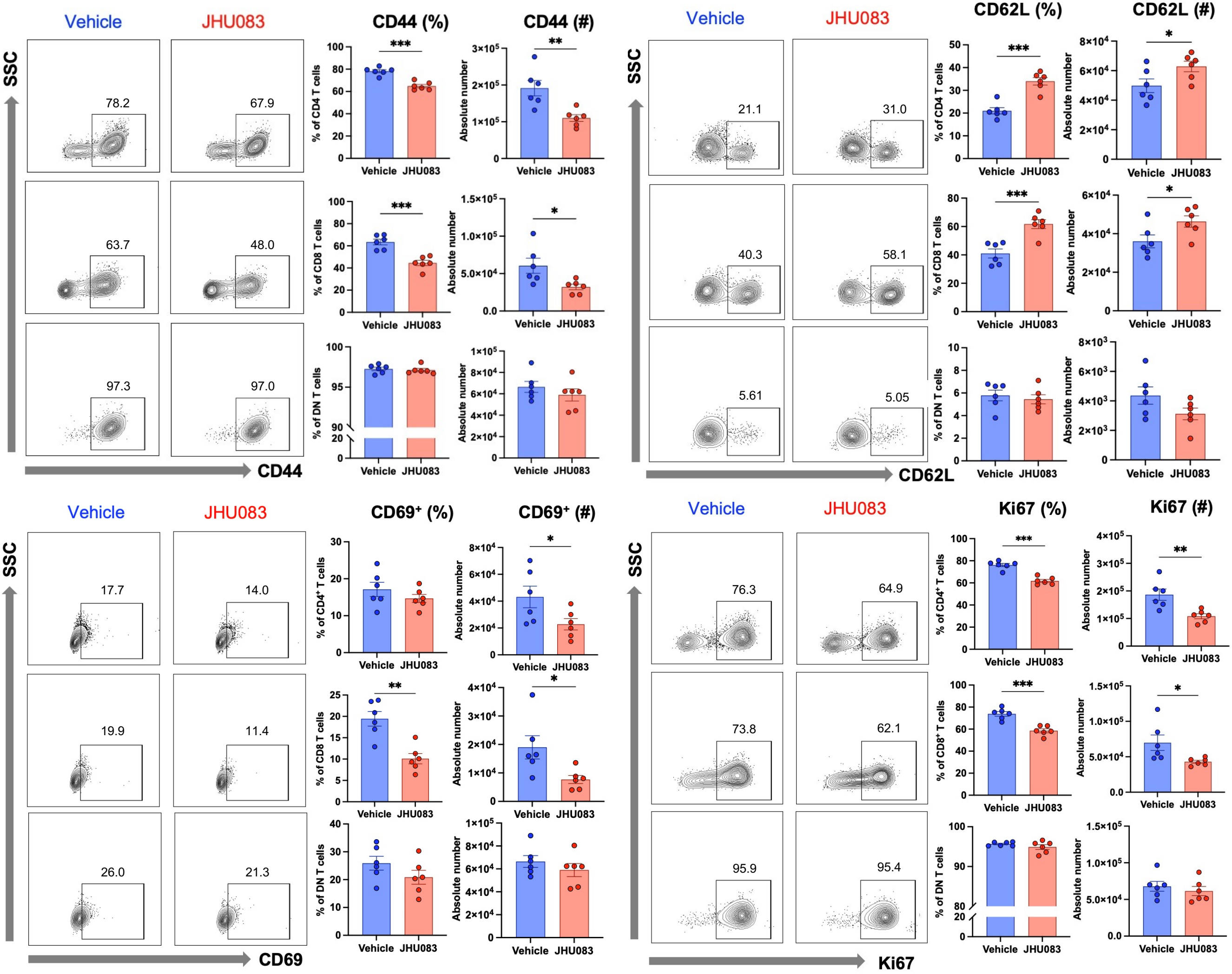

Supplemental Figure 6. Effects of glutamine blockade on T cell phenotypes in normal steady state kidneys. JHU083 treatment reduced CD44 expression and increased CD62L expression on $CD4^{+}$ and CD8 T^{+} cells in normal kidneys, indicating glutamine blockade reduced effector-memory phenotypes. CD69 expression was reduced in $CD8^{+}$ T cells from the JHU083 treated mice. Ki67 expression was reduced in $CD4^{+}$ and $CD8^{+}$ T cells from JHU083 treated normal kidneys. Statistical analyses were performed using two-tailed *t* test (*n* = 6 mice in each group). . $^{*}P < 0.05$; $^{**}P < 0.01$; $^{***}P < 0.001$.

DN, double-negative.
